# Rewiring of Cellular Division Site Selection in Evolution of Fission Yeasts

**DOI:** 10.1016/j.cub.2015.02.056

**Published:** 2015-05-04

**Authors:** Ying Gu, Candice Yam, Snezhana Oliferenko

**Affiliations:** 1 Randall Division of Cell and Molecular Biophysics, King’s College London, London SE1 1UL, UK; 2 Institute of Molecular and Cell Biology, 61 Biopolis Drive, Singapore 138673, Singapore; 3 Department of Biological Sciences, National University of Singapore, Singapore 117543, Singapore

## Abstract

Strategies to position the division apparatus exhibit a bewildering diversity [1], but how these mechanisms evolve remains virtually unknown. Here, we explore the plasticity of division site positioning in fission yeasts *Schizosaccharomyces pombe* and *Schizosaccharomyces japonicus*. We demonstrate that, whereas both species divide in the middle, only *S. pombe* uses the anillin Mid1 as a primary nucleus-derived cue to assemble the actomyosin ring at the equatorial cortex. We trace this variance to the divergence in subcellular targeting of Mid1 and show that duplication of an ancestral anillin early in the *Schizosaccharomyces* lineage may have led to subfunctionalization of the Mid1 orthologs. In contrast to *S. pombe*, medial assembly of the actomyosin ring in mitotic *S. japonicus* relies on the cortical anchor protein Cdc15 regulated by the tip-localized kinase Pom1. Our data suggest that division site placement is determined by cortical positioning of the actomyosin-plasma membrane linkers and that both identity of the linker and control of its subcellular targeting are highly modular.

## Highlights

• Division site selection diverged in the fission yeasts *S. pombe* and *S. japonicus*
• Only *S. pombe* uses the nucleus-derived anillin Mid1 to position the actomyosin ring
• *S. japonicus* uses the F-BAR protein Cdc15 regulated by Pom1 to cue ring assembly
• Mid1 subfunctionalized in *Schizosaccharomyces* following anillin gene duplication

## Teaser

Using closely related yeasts, Gu et al. show that placement of the division apparatus is determined by positioning of the actomyosin-plasma membrane linkers and that both identity of the linker and control of its subcellular targeting are subject to evolutionary plasticity.

## Results and Discussions

**Figure 1:**
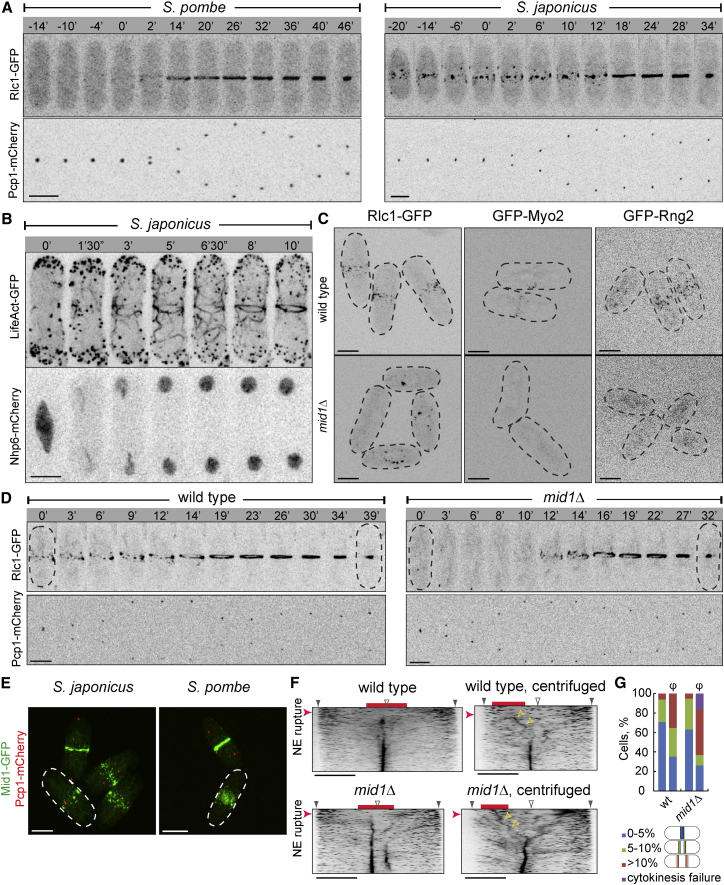
*S. japonicus* and *S. pombe* Exhibit Markedly Different Strategies of the Actomyosin Division Ring Assembly and Positioning (A) Time-lapse maximum-projection images of *S. pombe* (left) and *S. japonicus* (right) cells co-expressing the light chain of myosin II Rlc1-GFP and the SPB marker Pcp1-mCherry. (B) Time-lapse maximum-projection images of *S. japonicus* cell co-expressing LifeAct-GFP and the high-mobility group protein Nhp6-mCherry to mark nucleoplasm. (C) Maximum-projection images of interphase wild-type and *mid1*Δ *S. japonicus* cells expressing Rlc1-GFP, GFP-Myo2, and GFP-Rng2, respectively. Black dashed lines indicate cell boundaries. (D) Time-lapse maximum-projection images of mitotic wild-type (left) and *mid1*Δ (right) *S. japonicus* cells co-expressing Rlc1-GFP and Pcp1-mCherry. (E) Maximum-projection images of *S. japonicus* (left) and *S. pombe* (right) cells co-expressing Mid1-GFP and Pcp1-mCherry. White dashed lines mark the interphase cells. (F) Kymographs of cortical F-actin dynamics in mitotic LifeAct-GFP Nhp6-mCherry expressing wild-type (top) and *mid1*Δ (bottom) *S. japonicus* cells without (left) or with (right) nuclear displacement by centrifugation. Time interval is 10 s. Red bars indicate nuclear position. Red arrowheads indicate the time frame when the NE ruptures. Yellow arrowheads indicate trajectories of F-actin repositioning over time. Grey wedges mark the cell tips. Hollow wedges indicate cellular equator. (G) Graph representing proportions of wild-type and *mid1*Δ *S. japonicus* cells with septa at indicated cellular positions. Centrifuged samples are labeled as “φ.” Wild-type, n = 17 cells each for both experiments; *mid1*Δ, n = 19 cells each for both experiments. For (A), (B), and (D), time is in minutes and seconds. Scale bars represent 5 μm. See also Figure S1 and Movies S2 and S3.

In the fission yeast *S. pombe*, the myosin II localizes to the equatorial cortex at the G2/M transition, compacting into a medially positioned ring prior to anaphase. Ring constricts following chromosome segregation (Figures 1A and S1A; n = 10 cells; reviewed in [2]). In the related species *S. japonicus*, the myosin II marked by the regulatory light chain Rlc1-GFP formed an equatorial band of cortical nodes already in interphase. At the mitotic exit, the myosin nodes condensed into a ring that immediately constricted (Figures 1A and S1A; n = 25 cells). Following constriction, intracellular Rlc1-GFP gradually relocated to the cortex (Movie S1; the average time of cortical recruitment was 33.7 ± 14.2 min; n = 12 cells). Co-imaging with the F-actin marker LifeAct-mCherry revealed that the myosin II was indeed present at the cortex in interphase when most F-actin was associated with the growing cell tips (Figure S1B).

*S. japonicus* did not initiate equatorial actin assembly until late anaphase, consistent with early observations of fixed cells [3]. Again, ring formed only after mitotic spindle breakdown (Figure S1C). *S. japonicus* ruptures the nuclear envelope (NE) in late mitosis [4, 5]. Initial appearance of LifeAct-GFP-marked actin filaments at the medial cortex coincided with NE breakage (Figure 1B; n = 18 cells). In line with bipolar F-actin distribution, mitotic *S. japonicus* cells elongated until the actomyosin ring assembly (Figure S1C; 2 ± 1.1 μm/hr; n = 8 cells). Thus, the mode of cortical myosin II recruitment and the timing of actomyosin ring assembly diverged between the two species.

In *S. pombe*, the anillin Mid1 recruits the actomyosin to the cellular equator in early mitosis [6]. Cells lacking Mid1 delay actomyosin recruitment until the mitotic exit and misposition the division site [7–11]. Surprisingly, *mid1*Δ *S. japonicus* cells divided in the middle (Figure S1D). Removal of the second anillin Mid2 [12, 13] did not exacerbate the phenotype (Figures S1D and S1E). Suggesting that both anillins functioned in the later stages of cytokinesis, we observed many daughter cell pairs that remained connected at the septa and multiseptated cells in *mid1/2*Δ mutants (Figures S1D and S1E). Similarly to *S. pombe* [14–16], *mid2* transcript levels in *S. japonicus* peaked at mitosis, but *mid1* expression remained relatively constant throughout the cell cycle (Figure S1F).

The myosin II light (Rlc1) and heavy (Myo2) chains as well as the IQGAP protein Rng2 were delocalized from the cortex in interphase *mid1*Δ cells (Figure 1C; n > 25 cells). Yet, the actomyosin components were recruited to the cellular equator at the end of mitosis and assembled morphologically normal rings (Figure 1D; n = 10 cells). The average time of ring assembly in *mid1*Δ cells was 10.7 ± 3.6 min after SPB separation (n = 9 cells), similar to the wild-type (11.0 ± 1.3 min; n = 12 cells; p = 0.797; two-tailed t test). The average ring constriction time in *mid1*Δ cells was 14.8 ± 3.3 min (n = 14 cells), significantly faster than in the control (19 ± 1.9 min; n = 10 cells; p = 0.000816; two-tailed t test). We concluded that, whereas *S. japonicus* Mid1 serves as an interphase cortical anchor for myosin, it is dispensable for ring assembly at the cellular equator.

Mid1-GFP formed a band of equatorial nodes during interphase and was incorporated into a ring following mitosis (Figure 1E). The subcellular distribution of Mid1 in interphase *S. japonicus* differed dramatically from that in *S. pombe*, where Mid1 exhibited nuclear enrichment at this cell-cycle stage (Figure 1E; n = 57 cells) [9, 17]. Inspection of Mid1 amino acid sequences revealed that the polybasic stretch that functions both as the nuclear localization signal (NLS) and the membrane-anchoring domain in *S. pombe* [17, 18] diverged in *S. japonicus*, including a 17-amino-acid insertion (Figure S1G). In-frame removal of the *S. japonicus*-specific insertion did not delocalize the protein from the medial cortex (Figure S1H). However, incorporation of the SV40-derived NLS [19] or construction of the chimeric Mid1 carrying the *S. pombe* NLS-containing region triggered moderate nuclear accumulation of the modified proteins during interphase (Figure S1H).

The nucleus is dynamically positioned at the cell center in fission yeast [20], and its displacement may constitute one of the major challenges to intracellular organization. We displaced the anaphase nucleus by centrifugation in wild-type and *mid1Δ S. japonicus* cells and visualized F-actin dynamics by LifeAct-GFP. In non-spun controls, F-actin appearing at the cortex following NE breakdown was efficiently captured at the cellular equator (Figure 1F; Movie S2). When the nucleus broke away from cell center, we observed an initial burst of actin polymerization at the cortex overlying the displaced nucleus (Figure 1F; Movie S2). The F-actin meshwork gradually remodeled, shifting toward the cell middle presumably through capture by the equatorially tethered myosin complex (Figure S1I). Suggesting that myosin could in turn remodel through interactions with actin filaments, we also observed rings that were mildly off-set in the direction of nuclear displacement (Figure 1G). The centrifuged *mid1Δ* cells frequently assembled such off-center rings or failed in ring assembly altogether, likely due to the lack of pre-existing equatorial myosin anchors (Figures 1F and 1G; Movie S3). Thus, it appears that the Mid1-dependent cortical band of myosin II could serve as a safeguard, ensuring that cytokinesis occurs at the cell middle.

**Figure 2:**
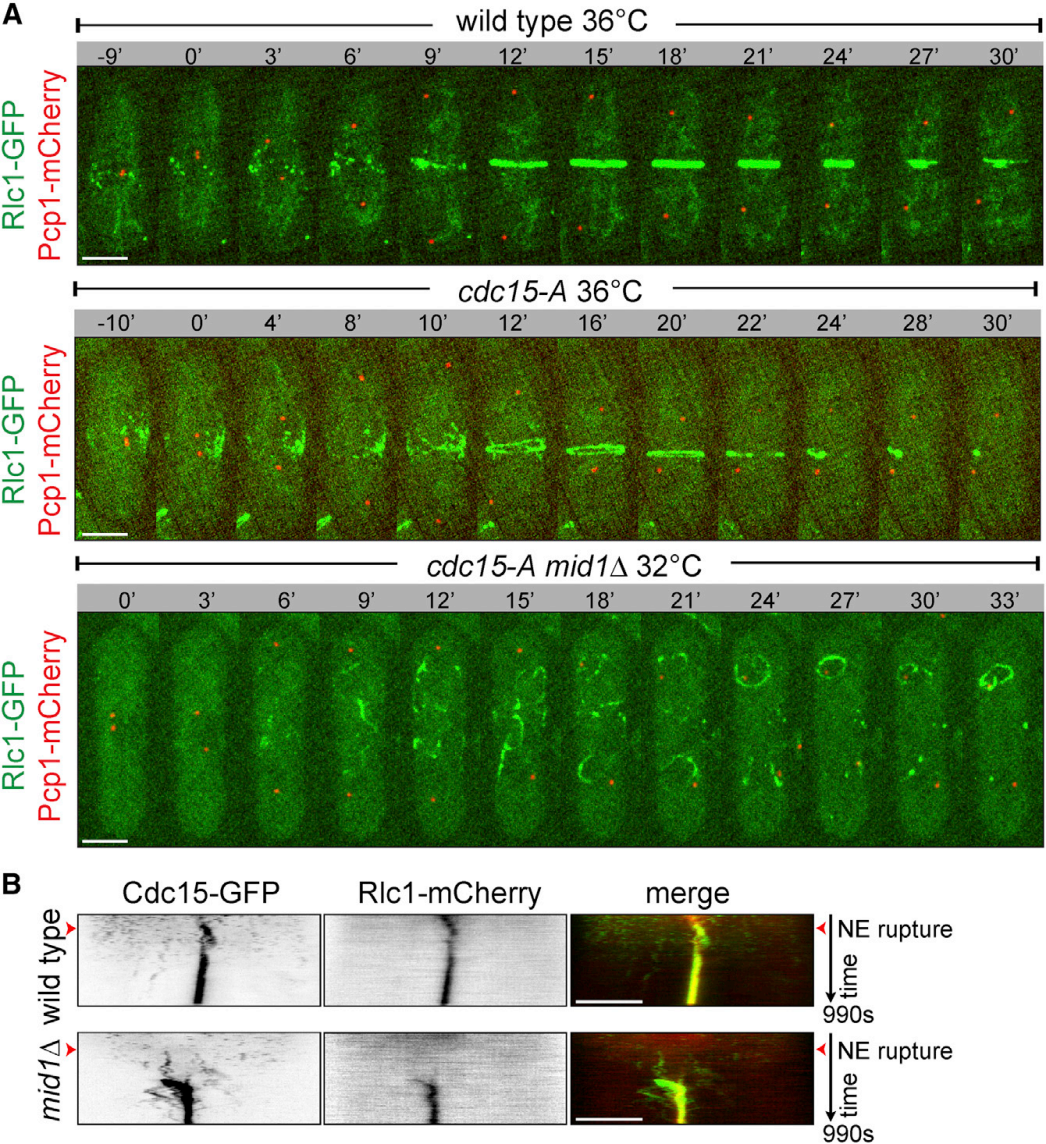
The F-BAR Protein Cdc15 Serves as a Primary Mitotic Anchor for the Actomyosin in *S. japonicus* (A) Time-lapse color-composite maximum-projection images of wild-type (top), *cdc15-A* temperature-sensitive mutant (middle), and *cdc15-Amid1*Δ double-mutant (bottom) *S. japonicus* cells expressing Rlc1-GFP and Pcp1-mCherry after incubation at indicated temperatures for 1 hr. Time is in minutes. (B) Kymographs showing dynamics of Cdc15-GFP and Rlc1-mCherry in mitotic wild-type (top) and *mid1*Δ (bottom) *S. japonicus* cells. Time interval is 10 s. Scale bars represent 5 μm. See also Figure S2.

The fact that myosin is recruited to the equatorial cortex during cytokinesis in *S. japonicus* cells lacking the interphase scaffold Mid1 suggested the existence of other cortical actomyosin anchor(s). In *S. pombe*, the F-BAR protein Cdc15 is thought to provide cortical linkages for the constricting ring [21–23]. We constructed a temperature-sensitive allele of *cdc15* in *S. japonicus*. At the restrictive temperature of 36°C, mutant *cdc15-A* cells exhibited medial myosin nodes that coalesced into a weakly fluorescent ring-like structure (Figure 2A; Rlc1-GFP fluorescence intensity in *cdc15-A* rings was 18% ± 4.1% of control; n = 8 cells). These structures fragmented soon after their formation, leading to cytokinesis failure (Figure S2A). The double-mutant *cdc15-Amid1Δ* cells failed in ring formation already at the semi-permissive temperature of 32°C. In these cells, myosin fibers that appeared at the end of mitosis were found throughout the cellular volume (Figure 2A). We concluded that simultaneous disruption of Mid1 and Cdc15 function abrogated cortical anchorage of the myosin II in *S. japonicus*.

In interphase *S. japonicus*, Cdc15-mCherry localized to the cell tips and also formed clusters at the medial cortex, partially co-localizing with Mid1-GFP. During mitosis, Cdc15 increasingly accumulated at Mid1 nodes at the cellular equator (Figure S2B). Although Cdc15-GFP did not localize to the medial cortex in interphase *mid1*Δ cells, it appeared there at the end of mitosis and was incorporated into cytokinetic rings (Figures 2B, S2C, and S2D).

**Figure 3:**
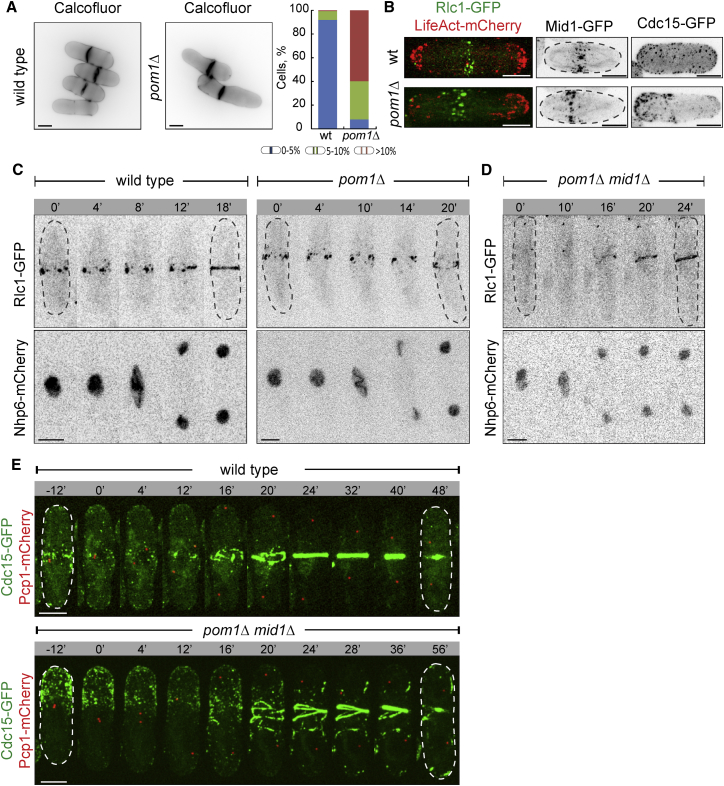
*S. japonicus* Cells Lacking the Polarisome Kinase Pom1 Misposition the Division Site (A) Calcofluor staining of wild-type and *pom1*Δ *S. japonicus* cells. Plot (right) shows proportion of cells exhibiting division septa at various positions along the long cell axis. 150 cells were counted for each genotype. (B) Maximum-projection images of interphase wild-type (top) and *pom1*Δ (bottom) *S. japonicus* cells expressing Rlc1-GFP and LifeAct-mCherry, Mid1-GFP, and Cdc15-GFP. The monopolar growth pattern in *pom1*Δ cells is revealed by LifeAct-mCherry. (C) Time-lapse maximum-projection images of wild-type (left) and *pom1*Δ (right) *S. japonicus* cells co-expressing Rlc1-GFP and Nhp6-mCherry. (D) A montage of time-lapse maximum-projection images of *pom1*Δ*mid1*Δ *S. japonicus* cells co-expressing Rlc1-GFP and Nhp6-mCherry. (E) Time-lapse maximum-projection images of wild-type (top) and *pom1*Δ*mid1*Δ (bottom) *S. japonicus* cells co-expressing Cdc15-GFP and Pcp1-mCherry (n = 6 cells). Dashed lines indicate cell boundaries. For (C)–(E), time is in minutes. Scale bars represent 5 μm. See also Figure S3.

In *S. pombe*, Pom1 kinase is thought to function in determining the cellular division site by inhibiting septum assembly at cell tips [24] and restricting Mid1 to the equatorial cortex during interphase [25, 26]. Pom1-GFP in interphase *S. japonicus* was enriched at the cell tips, similar to *S. pombe* (Figure S3A). *S. japonicus* cells lacking Pom1 were predominantly monopolar, as shown by phalloidin detection of F-actin (81% *pom1*Δ cells were monopolar as compared to 25% wild-type; n = 170 cells). Majority of *pom1*Δ cells divided off-center (Figure 3A), but the actomyosin rings were largely orthogonal (only 2.4% cells exhibited slightly tilted rings; n = 160 cells).

During interphase, the cortical myosin band was wider as compared to the wild-type (3.94 ± 1.28 μm versus 3.05 ± 0.95 μm for *pom1*Δ and wild-type, respectively; n = 40 cells; p = 0.000196; two-tailed t test) and displaced from the cellular equator toward a non-growing cell tip (Figure 3B; n = 27 out of 34 cells). Cortical myosin nodes exhibited mobility profiles comparable to control (Figure S3B). Mid1-GFP, Cdr2-GFP, and mCherry-Rng2 also relocalized away from cell equator (Figures 3B and S3C). Cdc15-GFP was found in a sock-like pattern at non-growing tips of interphase *pom1*Δ cells (Figure 3B). This was surprising because, in *S. pombe*, Cdc15 partitions to the cortical actin-rich domains active in endocytosis [27, 28]. Indeed, Cdc15 was enriched at the growing tip in *pom1*Δ *S. pombe* cells (Figure S3D).

During division of *pom1*Δ cells, the off-center actomyosin rings assembled slower than in the wild-type (18.3 ± 4.3 min versus 11.0 ± 1.3 min for *pom1*Δ [n = 14 cells] and the control [n = 12 cells], respectively; p = 1.73E−05; two-tailed t test). Ring constriction also occurred at a decreased rate (25.4 ± 5.5 min; n = 7 cells; p = 0.0208; two-tailed t test). Ring misplacement lead to the birth of two unequally sized daughter cells (Figures 3C and S3E; 11 out of 14 cells divided asymmetrically).

Similarly to single *pom1*Δ cells, double-mutant *pom1*Δ*mid1*Δ *S. japonicus* also mispositioned the division site (Figures 3D and S3F; 9 out of 11 cells divided asymmetrically). Rings formed at the boundary of Cdc15-rich cortical domain at a non-growing tip of *pom1*Δ*mid1*Δ cells (Figure 3E). Suggesting that actin cytoskeleton-associated processes controlled Cdc15 localization together with Pom1, Cdc15-GFP localized throughout the cellular cortex in *pom1*Δ *S. japonicus* cells treated with the actin polymerization inhibitor latrunculin A (Figure S3G). We concluded that, in *S. japonicas*, Pom1 plays a role in positioning the actomyosin ring through regulating the subcellular distribution of the cortical anchor Cdc15.

**Figure 4:**
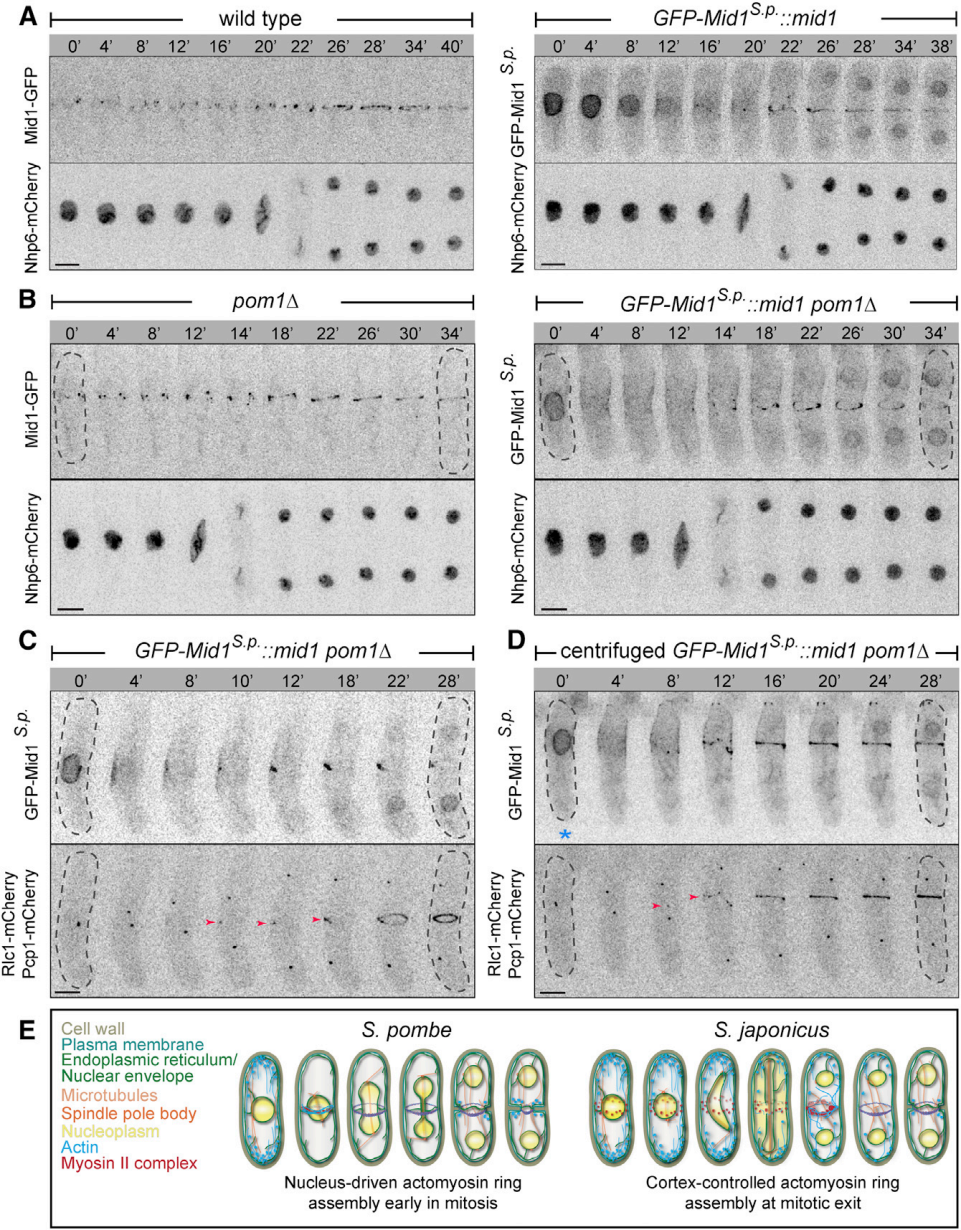
Replacement of *S. japonicus* Mid1 with Its *S. pombe* Ortholog Is Sufficient to Reconstruct Nucleus-Driven Division Site Positioning (A) Time-lapse maximum-projection images of *S. japonicus* cells co-expressing the endogenous GFP-tagged Mid1 (left) or its *S. pombe* ortholog GFP-Mid1^*S.p*.^ (right) together with the nuclear marker Nhp6-mCherry. (B) Time-lapse maximum-projection images of *pom1*Δ *S. japonicus* cells co-expressing either Mid1-GFP (left) or GFP-Mid1^*S.p*.^ (right) and Nhp6-mCherry. (C) Time-lapse maximum-projection images of *pom1*Δ *S. japonicus* cells co-expressing GFP-Mid1^*S.p*.^ with Rlc1-mCherry and the SPB marker Pcp1-mCherry to indicate mitotic progression. Dashed lines indicate cell boundaries. Red arrowheads indicate Rlc1-mCherry signal at the equatorial cortex. (D) Time-lapse maximum-projection images of *pom1*Δ *S. japonicus* cell co-expressing GFP-Mid1^*S.p*.^, Rlc1-mCherry and Pcp1-mCherry, where the nucleus was displaced toward a non-growing end by centrifugation. Dashed lines indicate cell boundaries. Red arrowheads indicate Rlc1-mCherry signal at the cortex. Blue asterisk indicates a growing cell tip. (E) Diagrams summarizing the modes of cytokinesis in *S. pombe* and *S. japonicus*. For (A)–(D), time is in minutes. Scale bars represent 5 μm. See also Figure S4.

In *S. pombe*, the centrally positioned nucleus promotes equatorial ring assembly early in mitosis by exporting nuclear Mid1 to the adjacent cortex [29]. We wondered whether replacing the *mid1* gene in *S. japonicus* with its *S. pombe* ortholog could re-establish the functional link between the nucleus and division site selection. The GFP-tagged Mid1^*S.pombe*^ (GFP-Mid1^*S.p.*^*)* knocked in into the native chromosomal locus localized to the nucleus in interphase *S. japonicus*. It redistributed to the lateral cortex early in mitosis (Figure 4A; n = 9 cells). Following the mitotic exit, GFP-Mid1^*S.p.*^ was incorporated into the actomyosin ring (Figure S4A; 43 out of 50 cells; see also Figure S4B). The timing of ring assembly (13.0 ± 3.0 min; n = 8 cells) and ring constriction (20.2 ± 4.1 min; n = 11 cells) in GFP-Mid1^*S.p.*^cells was comparable to the wild-type (p = 0.113 and 0.410, respectively; two-tailed t test). The nucleus-derived Mid1^*S.p*^ was effective in instructing an off-center actomyosin assembly in mitotic *S. japonicus* when nuclei were displaced toward cell tips by centrifugation (Figures S4C and S4D). We observed similar phenotype in cells where *S. japonicus* Mid1 was fused to the SV40-derived NLS (Figure S4D).

We then asked whether GFP-Mid1^*S.p.*^ could rescue the division-site-positioning defect associated with the lack of Pom1. Indeed, GFP-Mid1^*S.p.*^*pom1*Δ cells frequently assembled centrally positioned actomyosin rings (Figure 4B; n = 27 out of 42 cells). As expected from the exclusively nuclear localization of GFP-Mid1^*S.p.*^ during interphase, the myosin complex was not recruited to the medial cortex of GFP-Mid1^*S.p.*^ cells at this cell-cycle stage (Figure 4C; first time point). However, following Mid1^*S.p.*^ export from the nucleus, Rlc1-mCherry-marked myosin II colocalized with GFP-Mid1^*S.p.*^ at the cellular equator (Figure 4C; n = 11 out of 12 cells; see also Figure S4B). Thus, it appeared that Mid1^*S.p.*^ promoted medial actomyosin assembly in *S. japonicus*, possibly through providing early cortical anchor points for the myosin II and alleviating the Cdc15 cortical patterning defect associated with the loss of Pom1 function. Replacement of the *S. pombe* Mid1 with its *S. japonicus* counterpart virtually phenocopied *mid1* deletion (Figure S4E).

Shifting the anaphase nuclei away from cell center in GFP-Mid1^*S.p.*^*pom1*Δ cells led to two major functional outcomes. If the nucleus was displaced toward the non-growing tip, efflux of Mid1^*S.p.*^ from the nucleus early in mitosis promoted an off-center actomyosin ring assembly (Figure 4D; n = 12 cells). However, if the nucleus was shifted toward the growing tip, myosin failed to organize into a ring overlying the nucleus and instead formed long cable-like structures resulting in assembly of tilted and long axis septa (Figure S4F; six out of eight cells). Thus, polarized growth machinery appeared refractory to actomyosin ring anchorage, presumably through conflicted cortical patterning of the myosin II anchors Mid1 and Cdc15 in GFP-Mid1^*S.p.*^*pom1*Δ cells. Our results suggested that replacement of *S. japonicus* Mid1 with its *S. pombe* ortholog was sufficient to reconstruct the nucleus-instructive mode of ring assembly at the cellular equator.

Genomes of all fission yeasts encode two anillin-like proteins, orthologs of *S. pombe* Mid1 and Mid2 that in turn exhibit similarity to each other in the C-terminally located anillin homology domain and the PH-domain. Phylogenetic tree analysis of the fungal anillins revealed that genomes of every species with the exception of fission yeasts carried one anillin homolog clustering with Mid2 (Figure S4G). The genomes of other Taphrinomycotina [30] also carried a single *mid2*-like gene, suggesting that the Mid1-Mid2 pair could have arisen by duplication of a Mid2/Bud4-like protein in the ancestor of fission yeasts. Divergent regulation of gene expression in the duplicated pair may have driven early subfunctionalization (Figure S1F) [14–16, 31], with *S. pombe* eventually utilizing Mid1 for nucleus-dependent division site placement.

An evolutionarily conserved function for anillins appears to be scaffolding the cellular division machinery including actomyosin and septins [32–38]. Mid1 plays such structural role in *S. japonicus*, tethering the myosin II to the equatorial cortex in interphase and promoting recruitment of the mitotic anchor Cdc15 early in mitosis (Figures 1C, 2B, S2C, and S2D). Although the equatorial band of myosin is dispensable for medial division, it may enhance the fidelity of division site positioning upon insults to intracellular organization (Figures 1F and 1G).

As *S. japonicus* cells enter mitosis, the F-BAR protein Cdc15 takes over from Mid1 as a primary cortical tether for the actomyosin (Figure 2A). The key difference from *S. pombe* is that Cdc15 is able to localize to the cellular equator in the absence of Mid1 by utilizing signaling cues originating at the cell tips. Pom1 kinase and the actin-dependent growth machinery appear to function in specifying cortical distribution of Cdc15 (Figures 3 and S3G). Using cortical cues to control actomyosin tethering may allow *S. japonicus* to dynamically position the division site by reading out the state of the polarized growth machinery, rather than through tracking the nucleus. Unlike *S. pombe*, *S. japonicus* yeast cells readily transit to hyphal growth [39] and it would be of interest to determine whether controlling division by cortical signals provides physiological advantage in the context of a multicellular colony.

Unlike many cell types including *S. japonicus* and mammalian cells [1], *S. pombe* assembles the division rings early in mitosis [40]. Although the cortical actomyosin cables can be generated in *S. pombe mid1*Δ cells, they do not compact into orthogonal rings. Arguing for the lack of temporal coordination between actomyosin contractility and activation of septum assembly, inhibition of septum deposition allows *mid1*Δ cells sufficient time to correct ring orientation [11]. An interesting possibility is that precocious Mid1-dependent ring assembly may have arisen in *S. pombe* lineage to compensate for deregulation of the conventional, late mitotic pathway. An unrelated nuclear function in regulation of gene expression [41] could have promoted evolutionary co-option of Mid1 in instructive division site positioning in addition to its structural role in scaffolding cell division machinery.

The mitotic nucleus appears to influence ring assembly in *S. japonicus* by promoting actin polymerization. Possible signals could include local release of an activator of actin polymerization following NE breakdown or physical proximity to regulators localizing to the spindle or the SPBs.

Our work outlines a set of rules for generating functional diversity in division site placement (Figure 4E). Importantly, NE breakdown and post-mitotic assembly of a medial actomyosin ring position *S. japonicus* as a valuable genetically tractable model for understanding the logic of cytokinesis regulation used by higher eukaryotes.

## Experimental Procedures

### Yeast Strains and Culture Conditions

*S. japonicus* and *S. pombe* strains used in this study and their genotypes are listed in Table S1. *S. pombe* growth media and genetic methods were according to [42]. Auxotrophic heterothallic “wild-type” *S. japonicus* strains were a kind gift from H. Niki [43]. *S. japonicus* culture conditions and DNA transformation were as described in [44]. For details of strain construction, gene-expression analysis, and imaging methods, see the Supplemental Experimental Procedures.

## Supplemental Information

Document S1. Supplemental Experimental Procedures, Figures S1–S4, and Table S1

Movie S1. A Dividing Wild-Type *S. japonicus* Yields Two Daughter Cells, Related to the Results The myosin II complex is labeled by Rlc1-GFP, and the SPBs are marked by Pcp1-mCherry. Maximum projection of z-stacked spinning-disk confocal images is shown. Time is in minutes. The scale bar represents 5 μm.

Movie S2. Mitotic Wild-Type *S. japonicus* Cells Co-expressing LifeAct-GFP and Nhp6-mCherry, Related to Figure 1 Left panel: control. Right panel: a cell where the anaphase nucleus was displaced by centrifugation. LifeAct-GFP is pseudo-colored in grey. Maximum projections of z-stacked spinning-disk confocal images are shown. Time is in seconds. Underlined time frames indicate the timing of NE breakage. The scale bar represents 5 μm.

Movie S3. Mitotic *mid1*Δ *S. japonicus* Cells Co-expressing LifeAct-GFP and Nhp6-mCherry, Related to Figure 1 Left panel: control. Right panel: a cell where the anaphase nucleus was displaced by centrifugation. LifeAct-GFP is pseudo-colored in grey. Maximum projections of z-stacked spinning-disk confocal images are shown. Time is in seconds. Underlined time frames indicate the timing of NE breakage. The scale bar represents 5 μm.

Document S2. Article plus Supplemental Information
