# Supplementary material for: Rewiring of Cellular Division Site Selection in Evolution of Fission Yeasts
Source: Curr Biol. 2015 May 4;25(9):1187–94. doi: 10.1016/j.cub.2015.02.056 (PMC4425460; doi:10.1016/j.cub.2015.02.056)
Supplement: Document S1. Supplemental Experimental Procedures, Figures S1–S4, and Table S1 [file mmc1.pdf]

Current Biology
Supplemental Information

Rewiring of Cellular Division Site Selection in Evolution of Fission Yeasts

Ying Gu, Candice Yam, and Snezhana Oliferenko

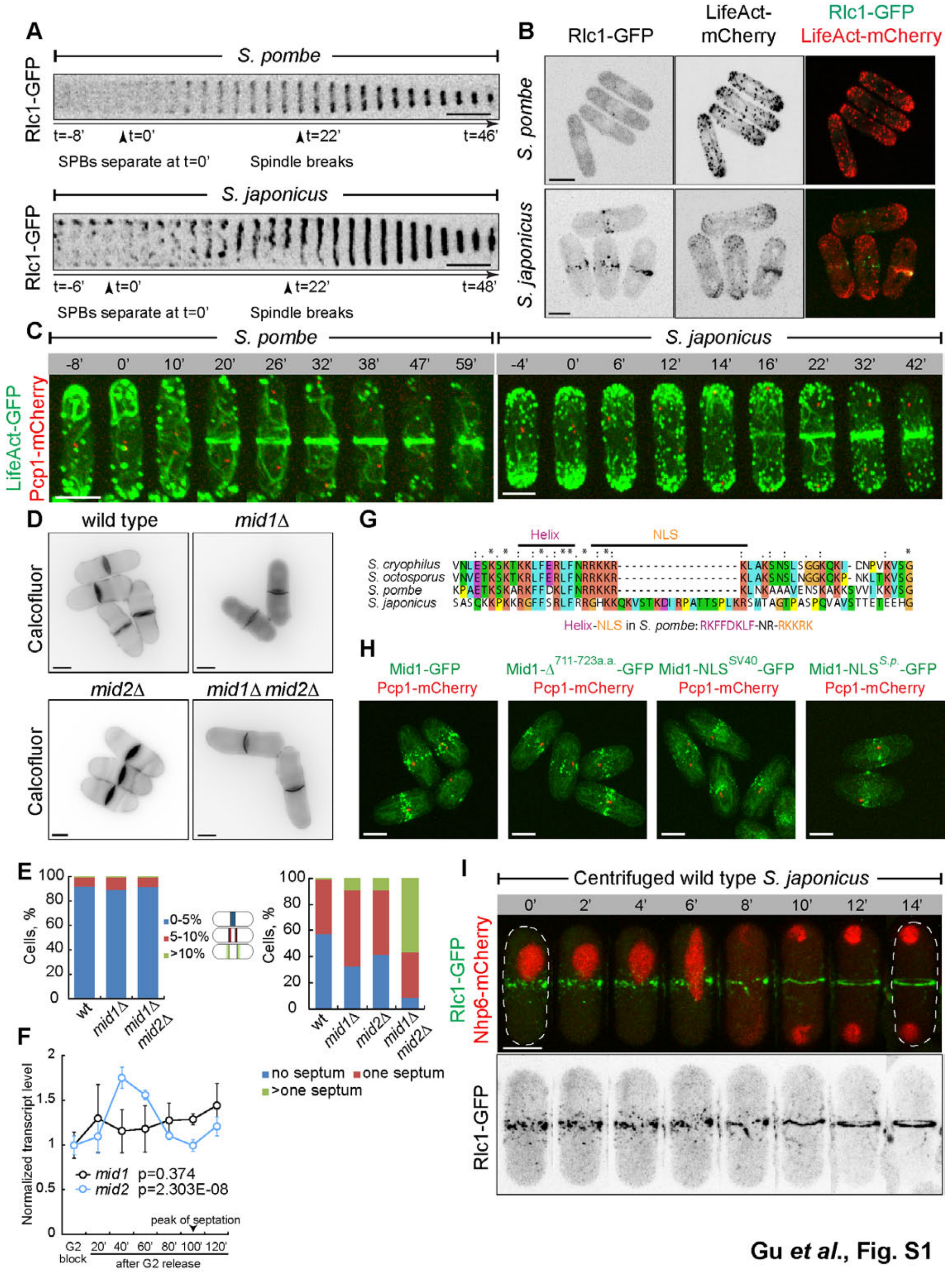

Gu *et al.*, Fig. S1

Figure S1, related to Figure 1

(**A**) Time-lapse maximum-projection images of Rlc1-GFP dynamics at the equatorial region of dividing *S. pombe* (top) and *S. japonicus* (bottom) cells. Mitotic progression has been monitored using Pcp1-mCherry-marked SPBs (see timeline underneath each montage). Time interval is 2 minutes. Arrowheads indicate reference time points. (**B**) Maximum-projection images of *S. pombe* (top) and *S. japonicus* (bottom) cells co-expressing Rlc1-GFP and the F-actin marker LifeAct-mCherry. Presented are individual channels and color composite images. The myosin II complex localizes to the medial cortex of interphase *S. japonicus* but not *S. pombe* cells. (**C**) Color composites of time-lapse maximum-projection images of *S. pombe* (left) and *S. japonicus* (right) cells co-expressing LifeAct-GFP and the SPB marker Pcp1-mCherry. n=13 cells for *S. pombe* and 25 cells for *S. japonicus*. (**D**) Calcofluor staining of wild type, *mid1*Δ, *mid2*Δ and *mid1*Δ*mid2*Δ *S. japonicus* cells. (**E**) Graph (left) showing proportion of cells exhibiting division septa at various positions along the long cell axis. Graph (right) showing fractions of cells with septa in asynchronously growing cell populations of indicated genotypes. We counted 151 wild type, 213 *mid1*Δ, 365 *mid2*Δ and 166 *mid1*Δ*mid2*Δ cells. (**F**) Normalized transcript levels of *mid1* and *mid2* at indicated time points in *cdc25-D9* block and release experiment. Error bars represent standard deviation. p-value estimated by ANOVA (one-way) test. (**G**) Sequence alignment of Mid1 proteins at the Helix-NLS region within the *Schizosaccharomyces* clade. (**H**) Color composites of maximum-projection images of *S. japonicus* cells co-expressing Mid1-GFP or indicated GFP-tagged Mid1 variants with the SPB marker Pcp1-mCherry. Mid1-GFP expressing cells, n=31; Mid1-$\Delta^{711\text{-}723a.a.}$-GFP cells, n=25. 83.3% of interphase Mid1-NLS^{SV40}-GFP cells (n=54) and 60% of interphase Mid1-$NLS^{S.p.}$-GFP cells (n=35) showed nuclear enrichment of the Mid1 mutant proteins. (**I**) Time-lapse maximum-projection images of a centrifuged *S. japonicus* cell co-expressing Rlc1-GFP and the nucleoplasm marker Nhp6-mCherry shown as the color composite (top) and the individual GFP channel (bottom). Dashed lines indicate cell boundaries. (**A**, **C**, **I**) Time is in minutes. Scale bars, 5 μm.

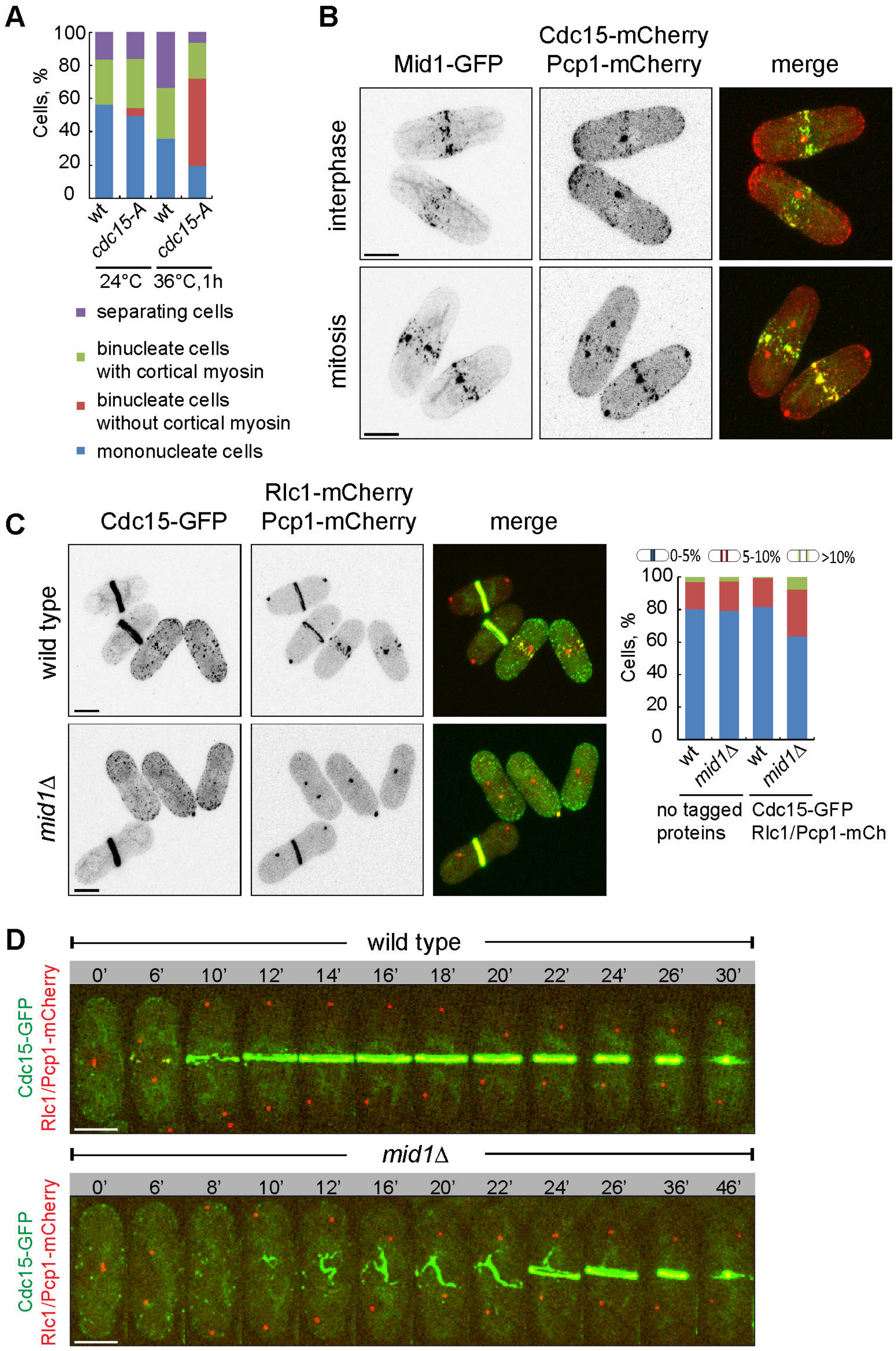

Gu *et al.*, Fig. S2

Figure S2, related to Figure 2

(**A**) Graph representing fractions of Rlc1-GFP-expressing cells exhibiting cell division phenotypes at the permissive temperature of 24°C or following incubation at the restrictive temperature of 36°C for 1 hour. Phenotypes were quantified in asynchronously growing cell populations. (**B**) Maximum-projection images of interphase and mitotic wild type *S. japonicus* cells co-expressing Mid1-GFP, Cdc15-mCherry and Pcp1-mCherry. Shown are individual channels and the composite color images. (**C**) Maximum-projection images of wild type (top) and *mid1*Δ (bottom) *S. japonicus* cells co-expressing Cdc15-GFP, Rlc1-mCherry and Pcp1-mCherry. Shown are individual channels and the composite color images. Of note, simultaneous presence of fluorophore-tagged Cdc15 and Rlc1 results in mild actomyosin ring positioning defects shown in the graph at far right. The graph represents fractions of Calcofluor-stained cells with septa at various cellular positions in asynchronously growing populations of indicated genotypes at 30°C. Wild type with no tagged proteins, n=210 cells; *mid1*Δ with no tagged proteins, n=195 cells; wild type with tagged proteins, n=206 cells; *mid1*Δ with tagged proteins, n=189 cells. (**D**) Color composites of time-lapse maximum-projection images of wild type (top) and *mid1*Δ (bottom) *S. japonicus* cells co-expressing Cdc15-GFP, Rlc1-mCherry and Pcp1-mCherry. Time is in minutes. Scale bars, 5 μm.

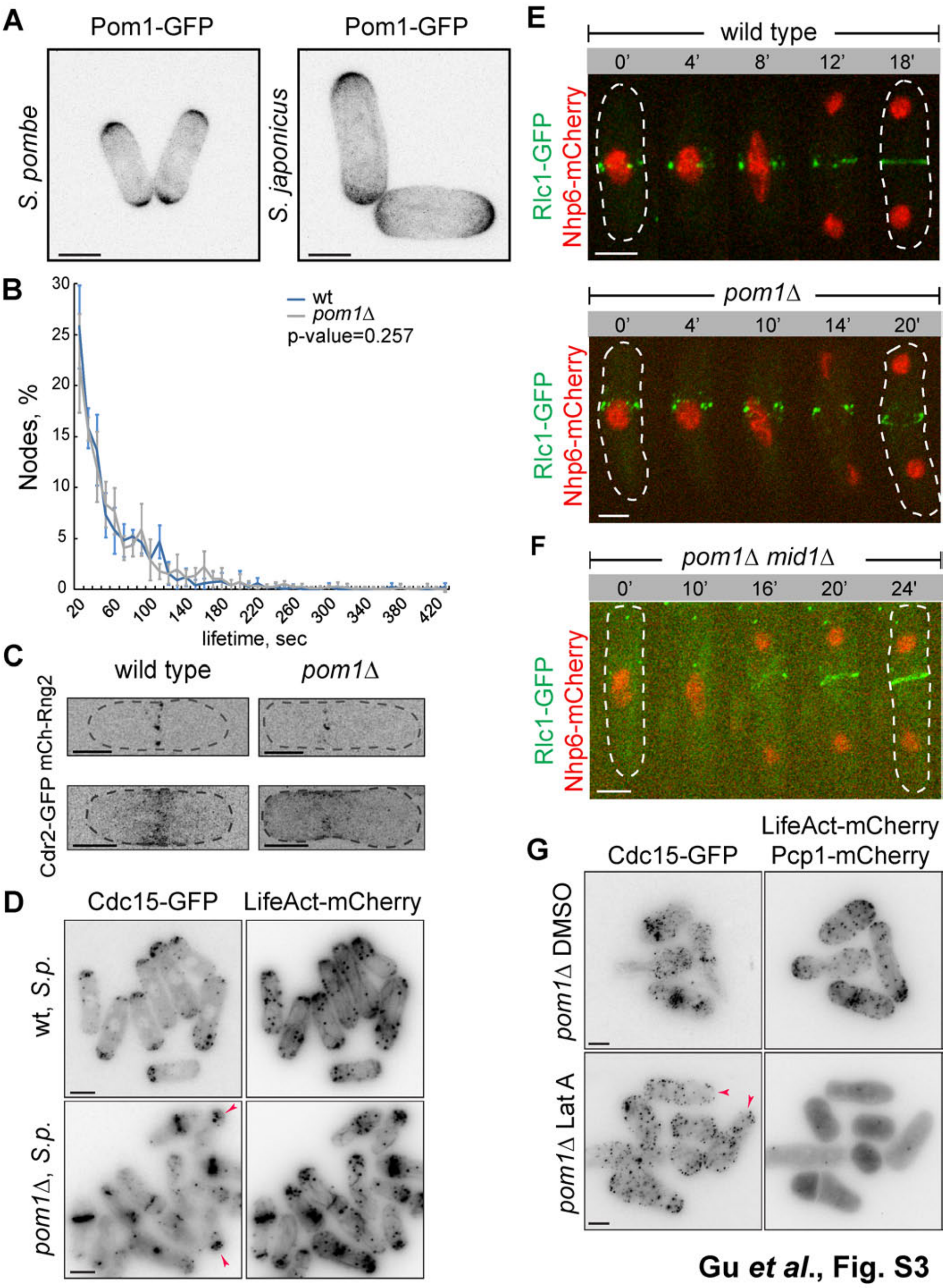

Gu *et al.*, Fig. S3

Figure S3, related to Figure 3

(**A**) Maximum-projection images of *S. pombe* (left) and *S. japonicus* (right) cells expressing Pom1-GFP. (**B**) Distribution of mobility profiles of interphase myosin nodes in wild type and *pom1Δ* cells, plotting the percentages of myosin nodes (Rlc1-GFP) with indicated lifetimes (dwelling times) at the medial cortex. n=5 cells. Error bars represent standard deviation. p-value estimated by two-sample Kolmogorov-Smirnov test. (**C**) Maximum-projection images of interphase wild type and *pom1*Δ *S. japonicus* cells expressing mCherry-Rng2 or Cdr2-GFP. (**D**) Maximum-projection images of wild type (top) and *pom1*Δ (bottom) *S. pombe* cells co-expressing Cdc15-GFP and LifeAct-mCherry. Shown are individual channels. Red arrowheads indicate the growing cell tip marked by LifeAct-mCherry. *S. p.*, *S. pombe*. (**E**) Color composites of time-lapse maximum-projection images of wild type (top) and *pom1*Δ (bottom) *S. japonicus* cells co-expressing Rlc1-GFP and Nhp6-mCherry, presented in Fig. 3C. (**F**) Color composites of time-lapse maximum-projection images of *pom1*Δ*mid1*Δ *S. japonicus* cell co-expressing Rlc1-GFP and Nhp6-mCherry, presented in Fig. 3D. (**G**) Maximum-projection images of *pom1*Δ *S. japonicus* cells co-expressing Cdc15-GFP, LifeAct-mCherry and Pcp1-mCherry treated with DMSO solvent (top) or LatrunculinA (bottom). Shown are individual channel images. (**E** and **F**) Dashed lines indicate cell boundaries. Time is in minutes. Scale bars, 5 μm .

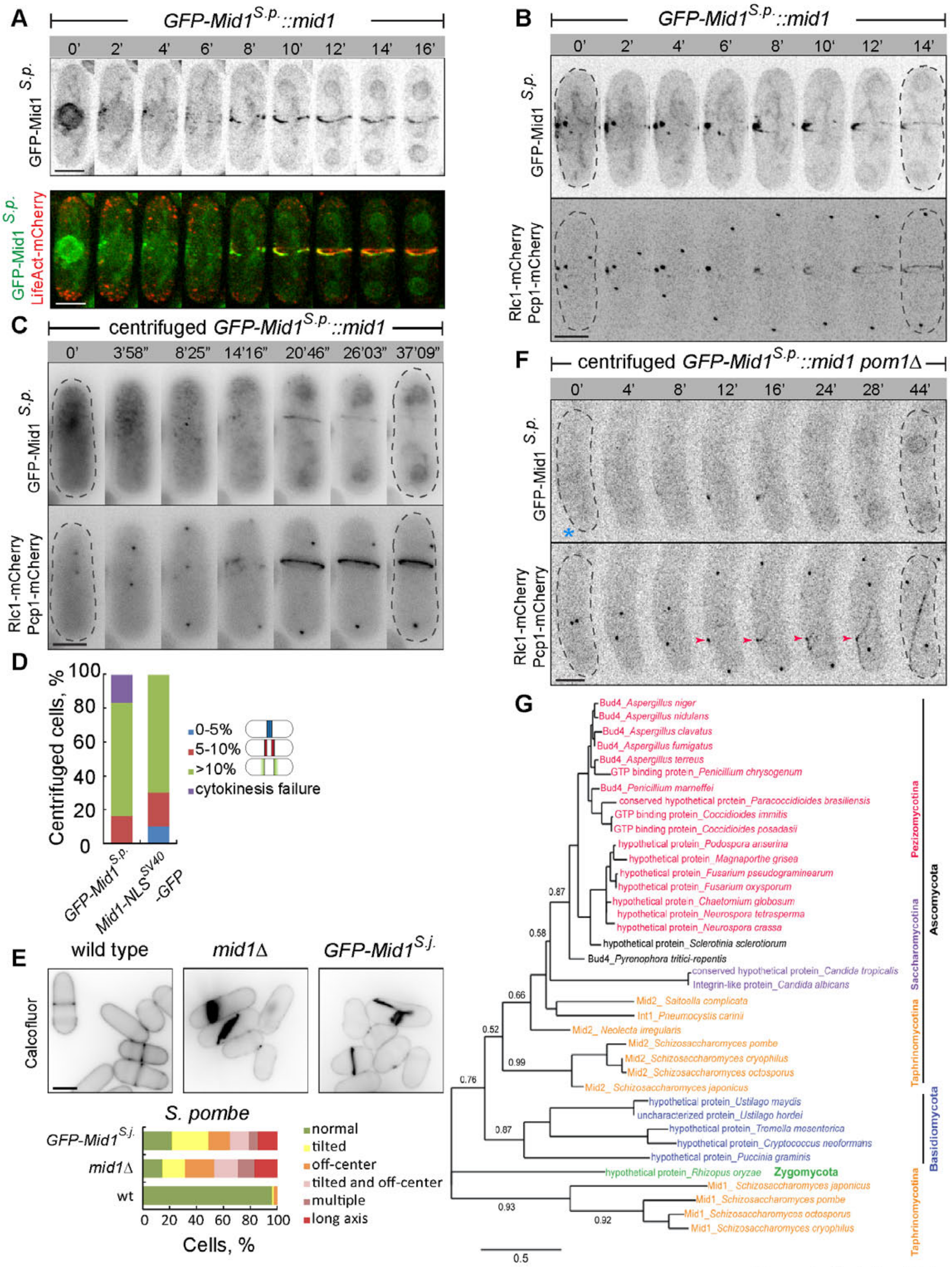

Gu *et al.*, Fig. S4

Figure S4, related to Figure 4

(**A**) Time-lapse maximum-projection images of *S. japonicus* co-expressing GFP-Mid1$^{S.p.}$ and LifeAct-mCherry show that GFP-Mid1$^{S.p.}$ is incorporated into the actomyosin division ring. Shown are the individual GFP channel (top) and the composite color (bottom) images. (**B**) Time-lapse maximum-projection images of *S. japonicus* co-expressing GFP-Mid1$^{S.p.}$ with Rlc1-mCherry and Pcp1-mCherry shows that the myosin II complex colocalizes with GFP-Mid1$^{S.p.}$ at the equatorial cortex early in mitosis. (**C**) Time-lapse single-plane images (upper panel for GFP, bottom panel for mCherry) of *S. japonicus* cell co-expressing GFP-Mid1$^{S.p.}$ with the myosin II light chain Rlc1-mCherry and the SPB marker Pcp1-mCherry, after nuclear displacement by centrifugation. (**D**) Graph representing proportions of GFP-Mid1$^{S.p.}$ and Mid1-NLS^{SV40}-GFP expressing *S. japonicus* cells with septa at indicated cellular positions after centrifugation. GFP-Mid1$^{S.p.}$, n=12 cells; Mid1-NLS^{SV40}-GFP, n=10 cells. (**E**) Calcofluor staining of *S. pombe* cells. Shown are the wild type (left), *mid1*Δ (center) and cells where the endogenous *mid1* has been replaced with its *S. japonicus* ortholog (right). Plot (below) shows proportion of cells exhibiting division septa at various positions along the long cell axis. Wild type cells, n=214; *mid1*Δ cells, n=221; GFP-Mid1$^{S.j.}$ cells, n=263. (**F**) Time-lapse maximum-projection images of *pom1*Δ *S. japonicus* cell co-expressing GFP-Mid1$^{S.p.}$ with the myosin II light chain Rlc1-mCherry and the SPB marker Pcp1-mCherry, where the nucleus was displaced towards a growing end by centrifugation. Note that Rlc1-mCherry fails to assemble into a ring. Red arrowheads indicate Rlc1-mCherry signal at the cortex. Blue asterisk indicates a growing cell tip. For (**A**-**C**, **F**), time is in minutes and seconds. Dashed lines indicate cell boundaries. Scale bars, 5 μm. (**G**) Phylogenetic tree of the anillin-like proteins in the fungal kingdom. A collection of protein sequences were pooled from representative species. Protein sequences were aligned and the phylogenetic tree was generated using Phylogeny (http://www.phylogeny.fr/).

Supplemental Table

Table S1. List of Strains

Schizosaccharomyces japonicus		
**Figure**	**Genotype**	**Collection No.**
1A, 1D, S1A, 2A	*Rlc1-GFP::kan^R Pcp1-mCherry::ura4+::kan^R ura4sj-D3 ade6sj-domE?*	SOJ1445
1B, 1F	*pAct1-Lifeact-GFP::ura4+ Nhp6-mCherry::ura4+ ura4sj-D3 ade6sj-domE?*	SOJ638
1C	*Rlc1-GFP::kan^R ura4sj-D3 h-*	SOJ27
	mid1Δ::ura4+ Rlc1-GFP::kan^R ura4sj-D3 ade6sj-domE?	SOJ1286
	GFP-Myo2::ura4+ura4sj-D3 ade6sj-domE h+	SOJ1556
	mid1Δ::kan^R GFP-Myo2::ura4+ura4sj-D3 ade6sj-domE?	SOJ1558
	GFP-Rng2::ura4 ura4sj-D3 ade6sj-domE h+	SOJ1518
	mid1Δ::kan^R GFP-Rng2::ura4+ ura4sj-D3 ade6sj-domE?	SOJ1539
1D	*mid1Δ::ura4+ Rlc1-GFP::kan^R Pcp1- mCherry::ura4+::kan^R ura4sj-D3 ade6sj-domE?*	SOJ1443
1E, S1H	*Mid1-GFP::ura4+::kan^R Pcp1-mCherry::ura4+::kan^R ura4sj-D3 ade6sj-domE*	SOJ1540
1F	*mid1Δ::ura4+ pAct1-Lifeact-GFP::ura4+ ura4sj-D3 ade6sj-domE*	SOJ2224
S1B, 3B	*Rlc1-GFP::kan^R pAct1-Lifeact-mCherry::ura4+ ura4sj-D3 ade6sj-domE?*	SOJ634
S1C	*pAct1-Lifeact-GFP::ura4+ Pcp1-mCherry::ura4+::kan^R ura4sj-D3 ade6sj-domE*	SOJ1481
S1D, 3A	*ura4sj-D3 ade6sj-domE h+*	SOJ88
S1D	*mid1Δ::ura4+ ura4sj-D3 ade6sj-domE h+*	SOJ680
	mid2Δ::kan^R ura4sj-D3 ade6sj-domE h+	SOJ1158
	mid1Δ::ura4+ mid2Δ::kan^R ura4sj-D3 ade6sj-domE	SOJ1299
S1F	*cdc25-D9::ura4+::kan^R ura4sj-D3 ade6sj-domE*	SOJ732
S1H	*Mid1-Δ$^{711-723a.a.}$-GFP::ura4+::kan^R Pcp1-mCherry::ura4+::kan^R ura4sj-D3 ade6sj-domE*	SOJ2208
	Mid1-NLS^{SV40}-GFP::ura4+::kan^R Pcp1-mCherry::ura4+::kan^R ura4sj-D3 ade6sj-domE	SOJ2207
	Mid1-NLS$^{S.p.}$-GFP::ura4+ Pcp1-mCherry::ura4+::kan^R ura4sj-D3 ade6sj-domE	SOJ2234
S1I, 3C, S3E	*Rlc1-GFP::kan^R Nhp6-mCherry::ura4+ ura4sj-D3 ade6sj-domE?*	SOJ694
2A	*cdc15-A::ura4+::kan^R Rlc1-GFP::kan^R Pcp1-mCherry::ura4+::kan^R ura4sj-D3 ade6sj-domE?*	SOJ2087
	cdc15-A::ura4+::kan^R mid1Δ::ura4+ Rlc1-GFP::kan^R Pcp1-mCherry::ura4+::kan^R ura4sj-D3 ade6sj-domE?	SOJ2213
2B	*Cdc15-GFP::ura4+::kan^R Rlc1-mCherry::ura4+::kan^R ura4sj-D3 ade6sj-domE*	SOJ2064
	mid1Δ::ura4+ Cdc15-GFP::ura4+::kan^R Rlc1-mCherry::ura4+::kan^R ura4sj-D3 ade6sj-domE	SOJ2062
S2B	*Mid1-GFP::ura4+::kan^R Cdc15-mCherry::ura4+::kan^R Pcp1-mCherry::ura4+::kan^R ura4sj-D3 ade6sj-domE*	SOJ2165
S2C, S2D	*Cdc15-GFP::ura4+::kan^R Rlc1-mCherry::ura4+::kan^R Pcp1-mCherry::ura4+::kan^R ura4sj-D3 ade6sj-domE*	SOJ2069
S2C, S2D	*mid1Δ::ura4+ Cdc15-GFP::ura4+::kan^R Rlc1-mCherry::ura4+::kan^R Pcp1-mCherry::ura4+::kan^R ura4sj-D3 ade6sj-domE*	SOJ2070
S3A	*Pom1-GFP::ura4+::kan^R ura4sj-D3 ade6sj-domE h+*	SOJ1339

3A	*pom1Δ::ura4+ ura4sj-D3 ade6sj-domE h+*	SOJ1031
3B, 4A	*Mid1-GFP::ura4+::kan^{R} Nhp6-mCherry::ura4+ ura4sj-D3 ade6sj-domE?*	SOJ1362
3B, 4B	*pom1Δ::ura4+ Mid1-GFP::ura4+::kan^{R} Nhp6-mCherry::ura4+ ura4sj-D3 ade6sj-domE?*	SOJ1400
3B, 3E	*Cdc15-GFP::ura4+::kan^{R} Pcp1-mCherry::ura4+::kan^{R} ura4sj-D3 ade6sj-domE*	SOJ2067
3B	*pom1Δ::ura4+ Rlc1-GFP::kan^{R} pAct1-Lifeact-mCherry::ura4+ ura4sj-D3 ade6sj-domE?*	SOJ1102
	pom1Δ::ura4+ Cdc15-GFP::ura4+::kan^{R} Pcp1-mCherry::ura4+::kan^{R} ura4sj-D3 ade6sj-domE	SOJ2161
3C, S3E	*pom1Δ::ura4+ Rlc1-GFP::kan^{R} Nhp6-mCherry::ura4+ ura4sj-D3 ade6sj-domE?*	SOJ1103
3D, S3F	*pom1Δ::ura4+ mid1Δ::ura4+ Rlc1-GFP::kan^{R} Nhp6-mCherry::ura4+ ura4sj-D3 ade6sj-domE?*	SOJ1586
3E	*pom1Δ::ura4+ mid1Δ::ura4+ Cdc15-GFP::ura4+::kan^{R} Pcp1-mCherry::ura4+::kan^{R} ura4sj-D3 ade6sj-domE*	SOJ2147
S3C	*mCherry-Rng2::ura4+ ura4sj-D3 ade6sj-domE h+*	SOJ1624
	pom1Δ::ura4+ mCherry-Rng2::ura4+ ura4sj-D3 ade6sj-domE	SOJ1658
	Cdr2-GFP::ura4+ ura4sj-D3 ade6sj-domE h+	SOJ974
	pom1Δ::ura4+ Cdr2-GFP::ura4+ ura4sj-D3 ade6sj-domE	SOJ1088
S3G	*pom1Δ::ura4+ Cdc15-GFP::ura4+::kan^{R} pAct1-Lifeact-mCherry::ura4+ Pcp1-mCherry::ura4+::kan^{R} ura4sj-D3 ade6sj-domE*	SOJ2162
4A	*GFP-Mid1$^{S.p.}$::ura4+::kan^{R} Nhp6-mCherry::ura4+ ura4sj-D3 ade6sj-domE?*	SOJ1449
4B	*pom1Δ::ura4+ GFP-Mid1$^{S.p.}$::ura4+::kan^{R} Nhp6-mCherry::ura4+ ura4sj-D3 ade6sj-domE?*	SOJ1447
4C, 4D, S4F	*pom1Δ::ura4+ GFP-Mid1$^{S.p.}$::ura4+::kan^{R} Rlc1-mCherry::ura4+::kan^{R} Pcp1-mCherry::ura4+::kan^{R} ura4sj-D3 ade6sj-domE*	SOJ1959
S4A	*GFP-Mid1$^{S.p.}$::ura4+::kan^{R} pAct1-Lifeact-mCherry::ura4+ ura4sj-D3 ade6sj-domE*	SOJ1451
S4B, S4C	*GFP-Mid1$^{S.p.}$::ura4+::kan^{R} Rlc1-mCherry::ura4+::kan^{R} Pcp1-mCherry::ura4+::kan^{R} ura4sj-D3 ade6sj-domE?*	SOJ1726
Schizosaccharomyces pombe		
**Figure**	**Genotype**	**Collection No.**
1A, S1A	*Rlc1-GFP::ura4+ Pcp1-mCherry::ura4+*	From M. Balasubramanian
S1B	*Rlc1-3GFP::kan^{R} pAct1-LifeAct-mCherry::leu1+*	From M. Balasubramanian
S1C	*pAct1-LifeAct-GFP::leu1+ Pcp1-mCherry::ura4+ leu1-32*	From M. Balasubramanian
1E	*Mid1-GFP::ura4+ Pcp1-mCherry::ura4+ ura4-D18 leu1-32 ade6*	SO3573
S3A	*Pom1-GFP::kan^{R}*	SO4594
S3D	*Cdc15-GFP::kan^{R} pAct1-LifeAct-mCherry::leu1+*	SO7955
	pom1Δ::ura4+ Cdc15-GFP::kan^{R} pAct1-LifeAct-mCherry::leu1+	SO7952
S4E	*ade6-M210 leu1-32 ura4-D18 h-*	SO1082
	mid1 Δ ::ura4+ leu1-32 ade6-M2x ura4-D18 h-	SO213
	GFP-Mid1$^{S.j.}$::his5+::ura4+::mid1 leu1-32 ade5D ade7::Ade5 ura4-D18 his5D	SO7810

Supplemental Experimental Procedures

Sequences and construction of strains

Fission yeast protein sequences were retrieved from the *Schizosaccharomyces* group database at the Broad Institute, USA (http://www.broadinstitute.org/annotation/genome/schizosaccharomyces_group/MultiHome.html) [S1]. Sequence alignment was performed using ClustalW2 [S2]. Phylogenetic trees were generated using Phylogeny (http://www.phylogeny.fr/) [S3]. To construct the LifeAct-GFP and LifeAct-mCherry *S. japonicus* strains, 1-kb PCR fragment of the *act1* promoter from *S. japonicus* was digested with ApaI and XhoI, and ligated into a pJK210-based pSO550 plasmid carrying the *S. japonicus ura4* gene as the selection marker. Nucleotide sequence encoding the LifeAct peptide was PCR amplified from the pJK148-Pact1-LAGFP plasmid, described in [S4]. LifeAct PCR fragment was joined to GFP or mCherry by fusion PCR. The LifeAct-GFP or LifeAct-mCherry fragment was double digested by XhoI and NotI and ligated into the pSO550 plasmid carrying the *act1* promoter. The final constructs pSO550-pAct1$^{S.japonicus}$-LifeAct-GFP/ mCherry were linearized with AfeI and transformed into yeast.

To generate gene knock-out strains in *S. japonicus*, we used either PCR-based homologous recombination strategy or the plasmid-based methodology. Briefly, primers harboring 80-bp flanking sequence of the target gene were used to amplify the KanMX6 cassette as the selection marker. The purified PCR product was directly used for yeast transformation. Alternatively, a pSO550 plasmid carrying the 5'UTR and 3'UTR fragments of the target gene, each approximately 500-bp in length, was linearized by appropriate enzymes and used for yeast transformation.

To generate the C-terminally tagged Rlc1-GFP strain, we used the PCR-based homologous recombination strategy as described in [S5]. Pcp1-mCherry, Mid1-GFP, Pom1-GFP, Cdc15-GFP, Cdc15-mCherry and Rlc1-mCherry were constructed according to the strategy described in [S6] with some modifications. Briefly, BglII- and NheI-digested PCR fragment of the KanMX6 cassette was used to replace the *S. japonicus ura4* cassette with the same flanking restriction enzyme sites as in the pSO550 plasmid. This construct was digested by BamHI and XbaI and ligated with the *S. japonicus ura4* cassette harboring the first 687 bp of *S. japonicus ura4* ORF. The resulting plasmid pKanMX6-Ura4ΔC$^{S. japonicus}$ was used to integrate the sequence encoding a C-terminally deleted *S. japonicus* Ura4 protein at the 3'UTR region of any target gene (creating an acceptor strain). A second pBluescriptKS-based plasmid was constructed to place the DNA sequence encoding a missing C-terminal fragment of S. japonicus Ura4 between BamHI and XbaI, to make the pKS-Ura4C$^{S. japonicus}$. DNA sequences encoding the C-terminus of any target protein fused to GFP or mCherry were cloned into pKS-Ura4C$^{S. japonicus}$ using ApaI and ClaI. The protein coding sequences were followed by ~200-bp 3'UTR of the target genes inserted between SmaI and BamHI sites. The resulting plasmid was used to PCR-amplify the region encoding the C-terminal target gene sequence followed by the fluorophore, the 3'UTR of the target gene and the Ura4 C-terminal fragment. This PCR product was used to transform the acceptor strain. Transformants were selected on the minimal medium plates without uracil. The GFP-Mid$^{S.pombe}$ *S.japonicus* strain was constructed similarly. As a result, the *S. japonicus mid1* promoter drove the expression of the N-terminally GFP-tagged *S. pombe* Mid1 that replaced the *S. japonicus mid1* ORF at its native locus.

To construct the N-terminally GFP- or mCherry-tagged Rng2 and Myo2 strains, the corresponding promoter sequences of *rng2* or *myo2* genes flanked by KpnI and ApaI sites were ligated into pSO550 plasmid carrying the *S. japonicus ura4* cassette. The obtained plasmids were then ligated to GFP- or mCherry-encoding sequences flanked by ApaI and XhoI, followed by the N-terminal ORF of Rng2 or Myo2 using XhoI and SmaI, and the adjacent genomic DNA fragments upstream to the promoter regions flanked by SmaI and BamHI. The obtained N-tagging plasmids were linearized by SmaI and transformed into yeast.

To create temperature sensitive mutants of *cdc25-D9* and *cdc15-A,* a PCR-based random mutagenesis strategy was adapted from [S6] with some modifications mentioned above. Acceptor strains carrying the C-terminally truncated *ura4* ORF cassette at the 3'UTR region of either *cdc25* or *cdc15* were generated using the plasmid pKanMX6-Ura4$\Delta C^{S. japonicus}$. Error-prone PCR products were amplified using the template plasmid pKS-Ura4$C^{S. japonicus}$ harboring a sequence of interest and used to transform the acceptor strain to reconstitute the *ura4+* cassette as the selective marker for successful integration. *cdc25-D9* carries four mutations in the ORF that change amino acid residues at positions 30 (Ile to Thr), 446 (Ile to Val), 494 (Thr to Ala) and 572 (Thr to Pro). *cdc15-A* allele carries two mutations in the encoded protein at positions 264 (Leu to Pro) and 442 (Pro to Thr).

To generate Mid1-NLS^{SV40}-GFP, a nucleotide sequence encoding the NLS (PKKKRKV) from the SV40 large T-antigen was inserted between codons corresponding to the amino acid position 725 and 726 of *S. japonicus mid1* ORF by fusion PCR. To construct Mid1-$NLS^{S.p.}$ –GFP, nucleotide sequence of *S. japonicus mid1* encoding amino acid residues from 650 to 760 was replaced with a coding region of *S. pombe mid1* spanning amino acid residues 639 to 730 by fusion PCR. To obtain Mid1-$\Delta^{711\text{-}723a.a.}$-GFP, a nucleotide sequence encoding the amino acid residues 711 to 723 of *S. japonicus mid1* ORF was deleted via fusion PCR.

mid1/mid2 transcription during cell cycle

Cell cycle synchronization of *S. japonicus* was performed using *cdc25-D9* conditional mutant. Briefly, *cdc25-D9* cells were grown in YES medium at 24°C to OD_{595} 0.2 and shifted to 36°C for 3 hours 30 minutes. Cells were collected and snap frozen at G2/M transition block point and at 20-minute intervals after lowering the temperature to 24°C. Total RNA was extracted using TRIzol® Reagent (Life Technologies). Transcriptor First Strand cDNA Synthesis Kit (Roche diagnostics) was used to synthesize cDNAs with anchored-oligo(dT)18 primer. PCR primers for real-time quantitative PCR (*mid1* 5'-TCCTGCGACAACTTCACCTC-3', 5'-GGCTTCCACCTGCTTCTCAT-3'; *mid2* 5'- CCATTCGCTCTTTCGGCATG-3', 5'- AGGTGTGTTGGCATCGTAGG-3'; *hcs1* 5'-TTTTCGAGCCTGGTATGCGT-3', 5'-CAGTCAAGGTTTGCTCACGC-3') were designed using Primer3 (Whitehead Institute, United States). Real-time quantitative PCR was performed with FastStart Essential DNA Green Master (Roche diagnostics). Data was collected and analyzed with LightCycler® 96 System (Roche diagnostics). *mid1/mid2* transcript levels were shown as relative linear ratio after normalization to *hcs1.*

Microscope image acquisition and data analyses

For time-lapse movies, mid-log phase cells were concentrated by centrifugation at 3,000 rpm for 30 sec and imaged on 2% agarose pads. Most imaging experiments were performed at 24ºC unless indicated otherwise. Epi-fluorescence images in Fig. 3A, S1D and S4E were captured using a Zeiss AxioVert200M microscope (Carl

Zeiss; Plan Apochromat 100×/1.45 NA oil objective lens) equipped with the CCD camera (CoolSNAP HQ; Photometrics) and driven by MetaMorph-v6.2r6 software package (Molecular Devices). Zeiss Axio Observer Z1 fluorescence microscope fitted with α Plan-FLUAR 100x/1.45 NA oil objective lens (Carl Zeiss) and the Orca-Flash4.0 C11440 camera (Hamamatsu) was used to acquire images shown in Fig. 2B, S3D, G and S4C. Scanning confocal images in Fig. 1C and S1B were acquired using a Nikon A1R Si Confocal system with Eclipse Ti-E Inverted microscope, Nikon Plan Apo VC 100x/NA1.4 oil objective, 457nm/488nm/514nm Argon, 561nm diode lasers, GaAsP Detectors, driven by NIS Elements C Scanning confocal images in Fig. 1E, 3C, S1(H, I), S2(B, C) and S3A were acquired using LSM510 microscope equipped with a Plan Apochromat 100X, 1.4 N.A. objective lens, a 488-nm argon laser and a 543-nm HeNe laser. Spinning-disk confocal images in Fig. 1(A, B and D), 3(C and D), 4(A-D), S1(A, C), S3(E, F). S4(A, B, F) were captured by the microLAMBDA system built on Eclipse Ti Nikon inverted microscope (Plan Apochromat VC 100×/1.40 NA oil objective lens) and equipped with CSUX1FW spinning disk head (Yokogawa Corporation of America), CCD camera (CoolSNAP HQ2), under the control of MetaMorph-v7.7.7.0 software. A 491-nm diode-pumped solid-state (DPSS) laser (Calypso) and 561-nm DPSS laser (Jive) were used for excitation. Spinning-disk confocal images in Fig. 2A, Fig. 3E and S2D were captured by Yokogawa CSU-X1 Spinning Disk Confocal with Eclipse Ti-E Inverted microscope with Nikon Plan Apo VC 100x/NA1.4 oil objective, 488nm diode, 561nm diode lasers and Andor Ixon3 EM-CCD camera controlled by NIS Elements AR. 0.5-μm step size images were typically collected.

When required, images were analyzed using ImageJ (National Institutes of Health). Ring assembly time was measured by time points spent between SPBs separation labeled by Pcp1-mCherry and fully formation of the actomyosin ring marked by Rlc1-GFP. Ring constriction time was shown as time points after the appearance of a fully formed ring till it completes constriction. Average ring assembly or constriction time was shown as mean±standard deviation. Kymographs were made with ImageJ plug-in KymoResliceWide developed by Eugene Katrukha.

Measurements of fluorescence intensity

Spinning-disk confocal images of Z-stacks with 0.5μm interval were processed with ImageJ using the sum projection method. For fluorescence intensity measurement of Rlc1-GFP, the mean intensity of an ROI created within any cellular boundary of the projected image was obtained and the background was background-subtracted. Fluorescence intensity was shown as mean±standard deviation.

Myosin nodes tracking

Time-lapse single plane images (10 sec/frame, 730 frames in total) of interphase wt and *pom1Δ* cells, carrying the myosin II light chain Rlc1-GFP and the SPB marker Pcp1-mCherry acquired with Zeiss observer epifluorescence microscope, were processed with TrackMate v2.3.0 (Fiji). Briefly, images were background-subtracted and then filtered using LoG detector, followed by LAP tracking with gap-closing, track splitting and merging functions. Tracking profile was further analyzed with Icy v1.5.2.0 (Quantitative Image Analysis Unit, Institut Pasteur) to obtain measurements shown in Fig. S3B. Only nodes that can be tracked progressively more than three consecutive frames were included.

LatrunculinA treatment

S. japonicus cells were treated with 10 μM LatrunculinA (Biomol International LP) for 15 minutes before imaging.

Cell centrifugation

All centrifugation experiments were done with a microcentrifuge (Heraeus Pico 17, Thermo Scientific) for 2 min at 13,300 rpm before imaging.

Supplemental References

S1. Rhind, N., Chen, Z., Yassour, M., Thompson, D.A., Haas, B.J., Habib, N., Wapinski, I., Roy, S., Lin, M.F., Heiman, D.I., et al. (2011). Comparative functional genomics of the fission yeasts. Science *332*, 930-936.

S2. Larkin, M.A., Blackshields, G., Brown, N.P., Chenna, R., McGettigan, P.A., McWilliam, H., Valentin, F., Wallace, I.M., Wilm, A., Lopez, R., et al. (2007). Clustal W and Clustal X version 2.0. Bioinformatics *23*, 2947-2948.

S3. Dereeper, A., Guignon, V., Blanc, G., Audic, S., Buffet, S., Chevenet, F., Dufayard, J.F., Guindon, S., Lefort, V., Lescot, M., et al. (2008). Phylogeny.fr: robust phylogenetic analysis for the non-specialist. Nucleic Acids Res *36*, W465-469.

S4. Huang, J., Huang, Y., Yu, H., Subramanian, D., Padmanabhan, A., Thadani, R., Tao, Y., Tang, X., Wedlich-Soldner, R., and Balasubramanian, M.K. (2012). Nonmedially assembled F-actin cables incorporate into the actomyosin ring in fission yeast. J Cell Biol *199*, 831-847.

S5. Bahler, J., Wu, J.Q., Longtine, M.S., Shah, N.G., McKenzie, A., 3rd, Steever, A.B., Wach, A., Philippsen, P., and Pringle, J.R. (1998). Heterologous modules for efficient and versatile PCR-based gene targeting in Schizosaccharomyces pombe. Yeast *14*, 943-951.

S6. Tang, X., Huang, J., Padmanabhan, A., Bakka, K., Bao, Y., Tan, B.Y., Cande, W.Z., and Balasubramanian, M.K. (2011). Marker reconstitution mutagenesis: a simple and efficient reverse genetic approach. Yeast *28*, 205-212.
