# Supplementary figures and images for: Rewiring of Cellular Division Site Selection in Evolution of Fission Yeasts
Source: Curr Biol. 2015 May 4;25(9):1187–94. doi: 10.1016/j.cub.2015.02.056 (PMC4425460; doi:10.1016/j.cub.2015.02.056)

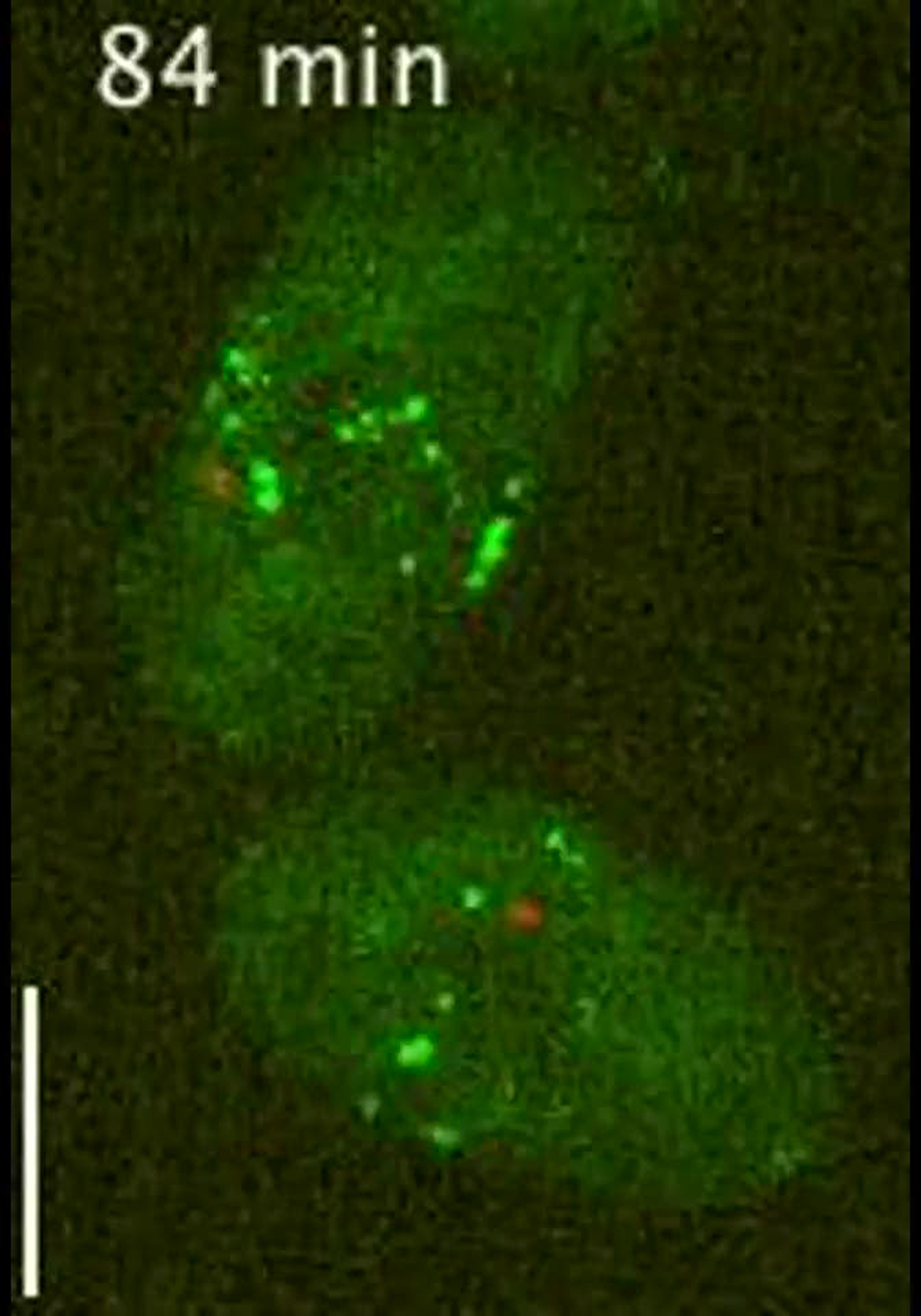

Supplement: Movie S1. A Dividing Wild-Type S. japonicus Yields Two Daughter Cells, Related to the Results — The myosin II complex is labeled by Rlc1-GFP, and the SPBs are marked by Pcp1-mCherry. Maximum projection of z-stacked spinning-disk confocal images is shown. Time is in minutes. The scale bar represents 5 μm. [file mmc2.jpg]

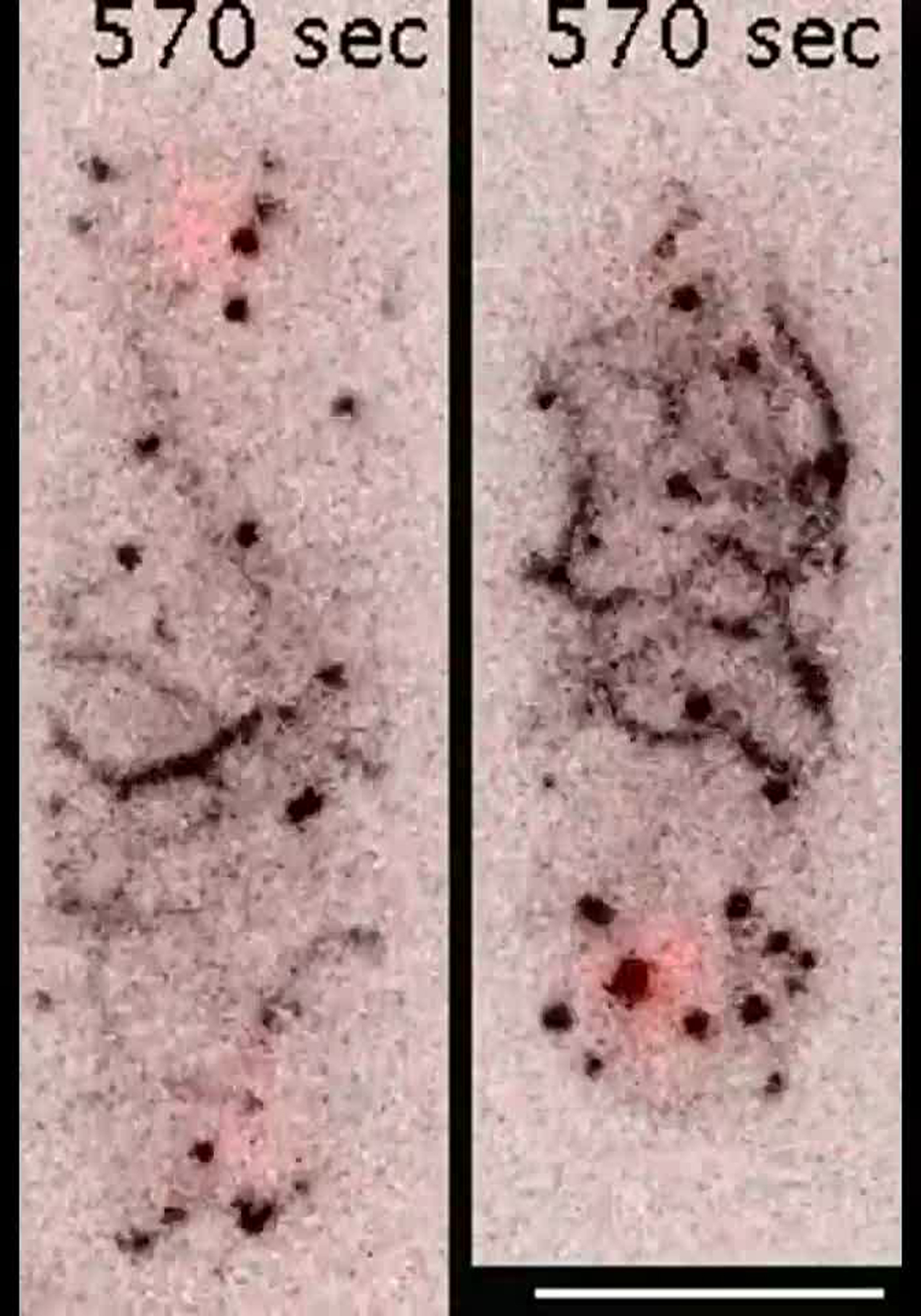

Supplement: Movie S2. Mitotic Wild-Type S. japonicus Cells Co-expressing LifeAct-GFP and Nhp6-mCherry, Related to Figure 1 — Left panel: control. Right panel: a cell where the anaphase nucleus was displaced by centrifugation. LifeAct-GFP is pseudo-colored in grey. Maximum projections of z-stacked spinning-disk confocal images are shown. Time is in seconds. Underlined time frames indicate the timing of NE breakage. The scale bar represents 5 μm. [file mmc3.jpg]

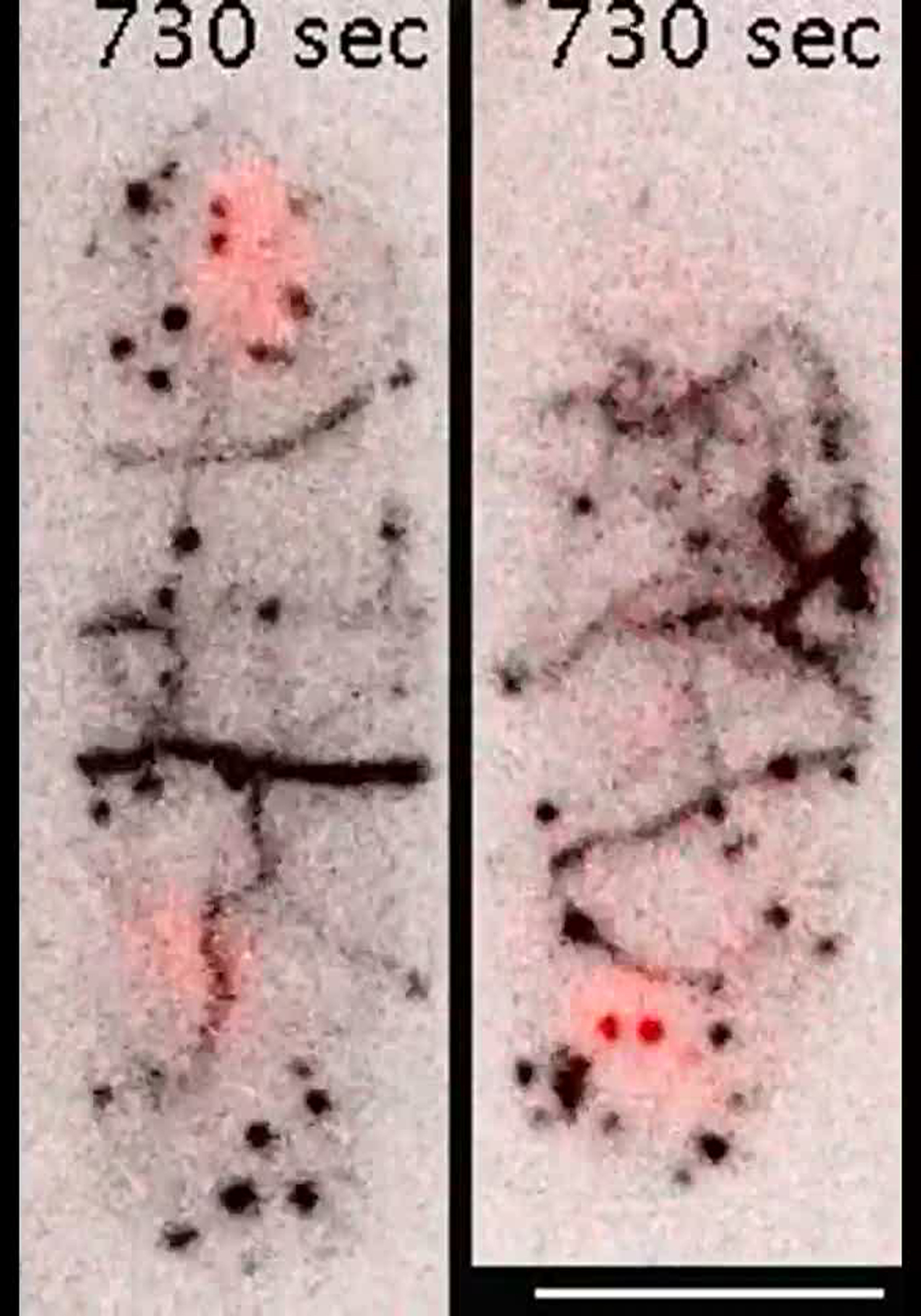

Supplement: Movie S3. Mitotic mid1Δ S. japonicus Cells Co-expressing LifeAct-GFP and Nhp6-mCherry, Related to Figure 1 — Left panel: control. Right panel: a cell where the anaphase nucleus was displaced by centrifugation. LifeAct-GFP is pseudo-colored in grey. Maximum projections of z-stacked spinning-disk confocal images are shown. Time is in seconds. Underlined time frames indicate the timing of NE breakage. The scale bar represents 5 μm. [file mmc4.jpg]
