# Supplementary material for: Rewiring of Cellular Division Site Selection in Evolution of Fission Yeasts
Source: Curr Biol. 2015 May 4;25(9):1187–94. doi: 10.1016/j.cub.2015.02.056 (PMC4425460; doi:10.1016/j.cub.2015.02.056)
Supplement: Document S2. Article plus Supplemental Information [file mmc5.pdf]

Report

Current Biology

Rewiring of Cellular Division Site Selection in Evolution of Fission Yeasts

Highlights

- Division site selection diverged in the fission yeasts *S. pombe* and *S. japonicus*
- Only *S. pombe* uses the nucleus-derived anillin Mid1 to position the actomyosin ring
- *S. japonicus* uses the F-BAR protein Cdc15 regulated by Pom1 to cue ring assembly
- Mid1 subfunctionalized in *Schizosaccharomyces* following anillin gene duplication

Authors

Ying Gu, Candice Yam, Snezhana Oliferenko

Correspondence

snezhana.oliferenko@kcl.ac.uk

In Brief

Using closely related yeasts, Gu et al. show that placement of the division apparatus is determined by positioning of the actomyosin-plasma membrane linkers and that both identity of the linker and control of its subcellular targeting are subject to evolutionary plasticity.

Gu et al., 2015, Current Biology *25*, 1187–1194
May 4, 2015 ©2015 The Authors
http://dx.doi.org/10.1016/j.cub.2015.02.056

Current Biology
Report

CellPress

Rewiring of Cellular Division Site Selection in Evolution of Fission Yeasts

Ying Gu,[1] Candice Yam,[2] and Snezhana Oliferenko[1,3,*]
[1]Randall Division of Cell and Molecular Biophysics, King's College London, London SE1 1UL, UK
[2]Institute of Molecular and Cell Biology, 61 Biopolis Drive, Singapore 138673, Singapore
[3]Department of Biological Sciences, National University of Singapore, Singapore 117543, Singapore
*Correspondence: snezhana.oliferenko@kcl.ac.uk
http://dx.doi.org/10.1016/j.cub.2015.02.056
This is an open access article under the CC BY license (http://creativecommons.org/licenses/by/4.0/).

SUMMARY

Strategies to position the division apparatus exhibit a bewildering diversity [1], but how these mechanisms evolve remains virtually unknown. Here, we explore the plasticity of division site positioning in fission yeasts *Schizosaccharomyces pombe* and *Schizosaccharomyces japonicus*. We demonstrate that, whereas both species divide in the middle, only *S. pombe* uses the anillin Mid1 as a primary nucleus-derived cue to assemble the actomyosin ring at the equatorial cortex. We trace this variance to the divergence in subcellular targeting of Mid1 and show that duplication of an ancestral anillin early in the *Schizosaccharomyces* lineage may have led to subfunctionalization of the Mid1 orthologs. In contrast to *S. pombe*, medial assembly of the actomyosin ring in mitotic *S. japonicus* relies on the cortical anchor protein Cdc15 regulated by the tip-localized kinase Pom1. Our data suggest that division site placement is determined by cortical positioning of the actomyosin-plasma membrane linkers and that both identity of the linker and control of its subcellular targeting are highly modular.

RESULTS AND DISCUSSIONS

In the fission yeast *S. pombe*, the myosin II localizes to the equatorial cortex at the G2/M transition, compacting into a medially positioned ring prior to anaphase. Ring constricts following chromosome segregation (Figures 1A and S1A; n = 10 cells; reviewed in [2]). In the related species *S. japonicus*, the myosin II marked by the regulatory light chain Rlc1-GFP formed an equatorial band of cortical nodes already in interphase. At the mitotic exit, the myosin nodes condensed into a ring that immediately constricted (Figures 1A and S1A; n = 25 cells). Following constriction, intracellular Rlc1-GFP gradually relocated to the cortex (Movie S1; the average time of cortical recruitment was 33.7 ± 14.2 min; n = 12 cells). Co-imaging with the F-actin marker LifeAct-mCherry revealed that the myosin II was indeed present at the cortex in interphase when most F-actin was associated with the growing cell tips (Figure S1B).

S. japonicus did not initiate equatorial actin assembly until late anaphase, consistent with early observations of fixed cells [3]. Again, ring formed only after mitotic spindle breakdown (Figure S1C). *S. japonicus* ruptures the nuclear envelope (NE) in late mitosis [4, 5]. Initial appearance of LifeAct-GFP-marked actin filaments at the medial cortex coincided with NE breakage (Figure 1B; n = 18 cells). In line with bipolar F-actin distribution, mitotic *S. japonicus* cells elongated until the actomyosin ring assembly (Figure S1C; 2 ± 1.1 μm/hr; n = 8 cells). Thus, the mode of cortical myosin II recruitment and the timing of actomyosin ring assembly diverged between the two species.

In *S. pombe*, the anillin Mid1 recruits the actomyosin to the cellular equator in early mitosis [6]. Cells lacking Mid1 delay actomyosin recruitment until the mitotic exit and misposition the division site [7–11]. Surprisingly, *mid1*Δ *S. japonicus* cells divided in the middle (Figure S1D). Removal of the second anillin Mid2 [12, 13] did not exacerbate the phenotype (Figures S1D and S1E). Suggesting that both anillins functioned in the later stages of cytokinesis, we observed many daughter cell pairs that remained connected at the septa and multiseptated cells in *mid1/2*Δ mutants (Figures S1D and S1E). Similarly to *S. pombe* [14–16], *mid2* transcript levels in *S. japonicus* peaked at mitosis, but *mid1* expression remained relatively constant throughout the cell cycle (Figure S1F).

The myosin II light (Rlc1) and heavy (Myo2) chains as well as the IQGAP protein Rng2 were delocalized from the cortex in interphase *mid1*Δ cells (Figure 1C; n > 25 cells). Yet, the actomyosin components were recruited to the cellular equator at the end of mitosis and assembled morphologically normal rings (Figure 1D; n = 10 cells). The average time of ring assembly in *mid1*Δ cells was 10.7 ± 3.6 min after SPB separation (n = 9 cells), similar to the wild-type (11.0 ± 1.3 min; n = 12 cells; $p = 0.797$; two-tailed t test). The average ring constriction time in *mid1*Δ cells was 14.8 ± 3.3 min (n = 14 cells), significantly faster than in the control (19 ± 1.9 min; n = 10 cells; $p = 0.000816$; two-tailed t test). We concluded that, whereas *S. japonicus* Mid1 serves as an interphase cortical anchor for myosin, it is dispensable for ring assembly at the cellular equator.

Mid1-GFP formed a band of equatorial nodes during interphase and was incorporated into a ring following mitosis (Figure 1E). The subcellular distribution of Mid1 in interphase *S. japonicus* differed dramatically from that in *S. pombe*, where Mid1 exhibited nuclear enrichment at this cell-cycle stage (Figure 1E; n = 57 cells) [9, 17]. Inspection of Mid1 amino acid sequences revealed that the polybasic stretch that functions

CrossMark

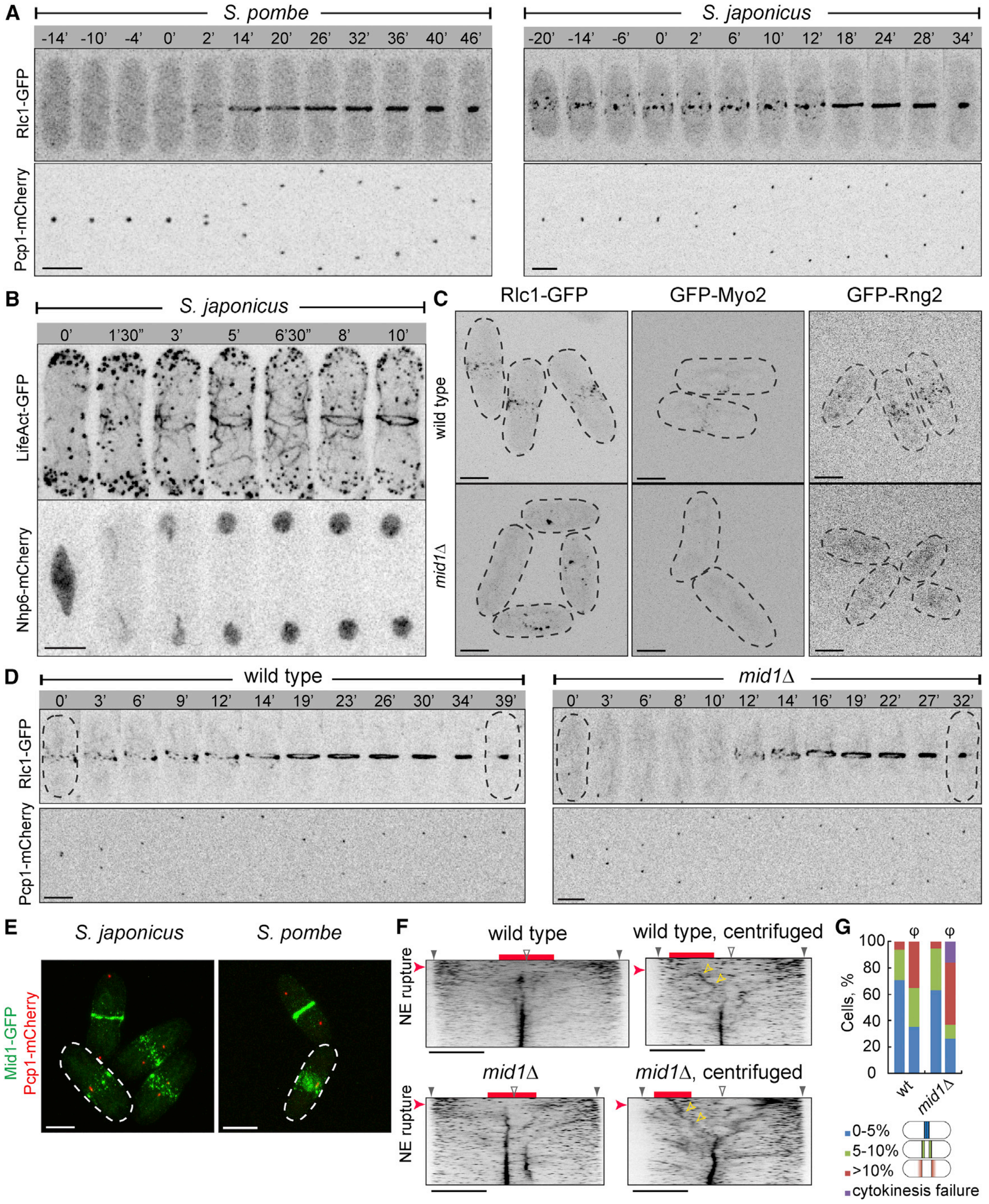

Figure 1. *S. japonicus* and *S. pombe* Exhibit Markedly Different Strategies of the Actomyosin Division Ring Assembly and Positioning

(A) Time-lapse maximum-projection images of *S. pombe* (left) and *S. japonicus* (right) cells co-expressing the light chain of myosin II Rlc1-GFP and the SPB marker Pcp1-mCherry.

(B) Time-lapse maximum-projection images of *S. japonicus* cell co-expressing LifeAct-GFP and the high-mobility group protein Nhp6-mCherry to mark nucleoplasm.

(C) Maximum-projection images of interphase wild-type and *mid1*Δ *S. japonicus* cells expressing Rlc1-GFP, GFP-Myo2, and GFP-Rng2, respectively. Black dashed lines indicate cell boundaries.

(D) Time-lapse maximum-projection images of mitotic wild-type (left) and *mid1*Δ (right) *S. japonicus* cells co-expressing Rlc1-GFP and Pcp1-mCherry.

(E) Maximum-projection images of *S. japonicus* (left) and *S. pombe* (right) cells co-expressing Mid1-GFP and Pcp1-mCherry. White dashed lines mark the interphase cells.

(F) Kymographs of cortical F-actin dynamics in mitotic LifeAct-GFP Nhp6-mCherry expressing wild-type (top) and *mid1*Δ (bottom) *S. japonicus* cells without (left) or with (right) nuclear displacement by centrifugation. Time interval is 10 s. Red bars indicate nuclear position. Red arrowheads indicate the time frame

(legend continued on next page)

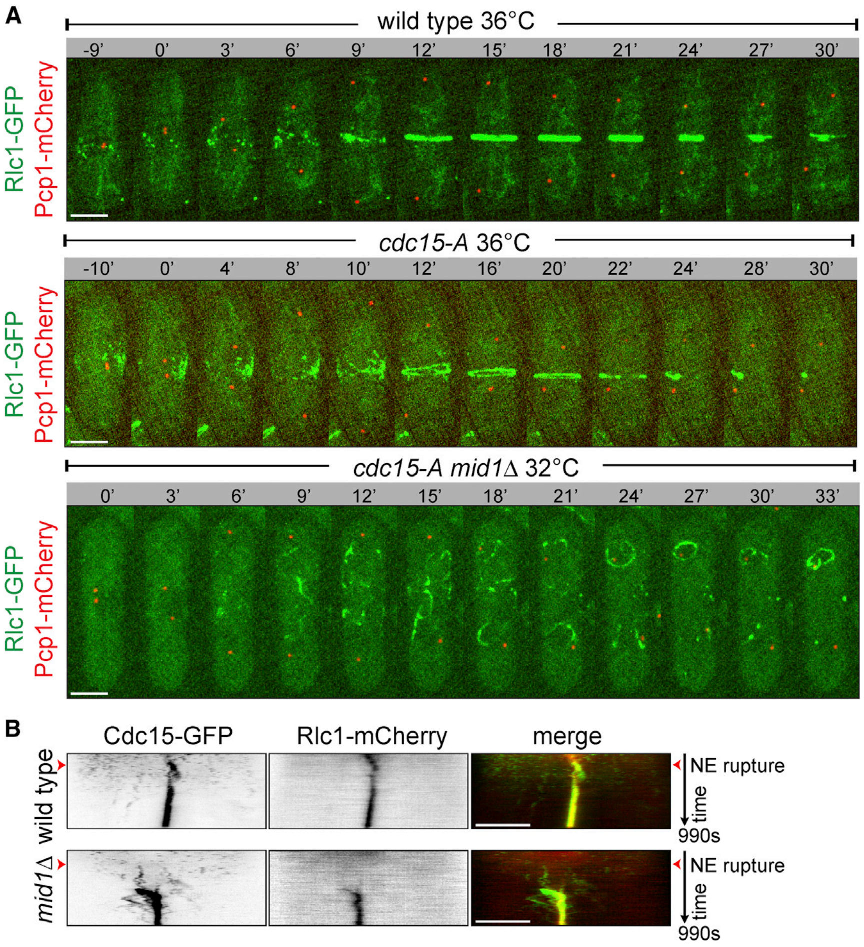

Figure 2. The F-BAR Protein Cdc15 Serves as a Primary Mitotic Anchor for the Actomyosin in *S. japonicus*

(A) Time-lapse color-composite maximum-projection images of wild-type (top), *cdc15-A* temperature-sensitive mutant (middle), and *cdc15-Amid1*Δ double-mutant (bottom) *S. japonicus* cells expressing Rlc1-GFP and Pcp1-mCherry after incubation at indicated temperatures for 1 hr. Time is in minutes.

(B) Kymographs showing dynamics of Cdc15-GFP and Rlc1-mCherry in mitotic wild-type (top) and *mid1*Δ (bottom) *S. japonicus* cells. Time interval is 10 s.

Scale bars represent 5 μm. See also Figure S2.

both as the nuclear localization signal (NLS) and the membrane-anchoring domain in *S. pombe* [17, 18] diverged in *S. japonicus*, including a 17-amino-acid insertion (Figure S1G). In-frame removal of the *S. japonicus*-specific insertion did not delocalize the protein from the medial cortex (Figure S1H). However, incorporation of the SV40-derived NLS [19] or construction of the chimeric Mid1 carrying the *S. pombe* NLS-containing region triggered moderate nuclear accumulation of the modified proteins during interphase (Figure S1H).

The nucleus is dynamically positioned at the cell center in fission yeast [20], and its displacement may constitute one of the major challenges to intracellular organization. We displaced the anaphase nucleus by centrifugation in wild-type and *mid1Δ* *S. japonicus* cells and visualized F-actin dynamics by LifeAct-GFP. In non-spun controls, F-actin appearing at the cortex following NE breakdown was efficiently captured at the cellular equator (Figure 1F; Movie S2). When the nucleus broke away from cell center, we observed an initial burst of actin polymerization at the cortex overlying the displaced nucleus (Figure 1F; Movie S2). The F-actin meshwork gradually remodeled, shifting toward the cell middle presumably through capture by the equatorially tethered myosin complex (Figure S1I). Suggesting that myosin could in turn remodel through interactions with actin filaments, we also observed rings that were mildly off-set in the direction of nuclear displacement (Figure 1G). The centrifuged *mid1Δ* cells frequently assembled such off-center rings or failed in ring assembly altogether, likely due to the lack of pre-existing equatorial myosin anchors (Figures 1F and 1G; Movie S3). Thus, it appears that the Mid1-dependent cortical band of myosin II could serve as a safeguard, ensuring that cytokinesis occurs at the cell middle.

The fact that myosin is recruited to the equatorial cortex during cytokinesis in *S. japonicus* cells lacking the interphase scaffold Mid1 suggested the existence of other cortical actomyosin anchor(s). In *S. pombe*, the F-BAR protein Cdc15 is thought to provide cortical linkages for the constricting ring [21–23]. We constructed a temperature-sensitive allele of *cdc15* in *S. japonicus*. At the restrictive temperature of 36°C, mutant *cdc15-A* cells exhibited medial myosin nodes that coalesced into a weakly fluorescent ring-like structure (Figure 2A; Rlc1-GFP fluorescence intensity in *cdc15-A* rings was 18% ± 4.1% of control; n = 8 cells). These structures fragmented soon after their formation, leading to cytokinesis failure (Figure S2A). The double-mutant *cdc15-Amid1Δ* cells failed in ring formation already at the semi-permissive temperature of 32°C. In these cells, myosin fibers that appeared at the end of mitosis were found throughout the cellular volume (Figure 2A). We concluded that simultaneous disruption of Mid1 and Cdc15 function abrogated cortical anchorage of the myosin II in *S. japonicus*.

when the NE ruptures. Yellow arrowheads indicate trajectories of F-actin repositioning over time. Grey wedges mark the cell tips. Hollow wedges indicate cellular equator.

(G) Graph representing proportions of wild-type and *mid1*Δ *S. japonicus* cells with septa at indicated cellular positions. Centrifuged samples are labeled as "φ." Wild-type, n = 17 cells each for both experiments; *mid1*Δ, n = 19 cells each for both experiments.

For (A), (B), and (D), time is in minutes and seconds. Scale bars represent 5 μm. See also Figure S1 and Movies S2 and S3.

In interphase *S. japonicus*, Cdc15-mCherry localized to the cell tips and also formed clusters at the medial cortex, partially co-localizing with Mid1-GFP. During mitosis, Cdc15 increasingly accumulated at Mid1 nodes at the cellular equator (Figure S2B). Although Cdc15-GFP did not localize to the medial cortex in interphase *mid1*Δ cells, it appeared there at the end of mitosis and was incorporated into cytokinetic rings (Figures 2B, S2C, and S2D).

In *S. pombe*, Pom1 kinase is thought to function in determining the cellular division site by inhibiting septum assembly at cell tips [24] and restricting Mid1 to the equatorial cortex during interphase [25, 26]. Pom1-GFP in interphase *S. japonicus* was enriched at the cell tips, similar to *S. pombe* (Figure S3A). *S. japonicus* cells lacking Pom1 were predominantly monopolar, as shown by phalloidin detection of F-actin (81% *pom1*Δ cells were monopolar as compared to 25% wild-type; n = 170 cells). Majority of *pom1*Δ cells divided off-center (Figure 3A), but the actomyosin rings were largely orthogonal (only 2.4% cells exhibited slightly tilted rings; n = 160 cells).

During interphase, the cortical myosin band was wider as compared to the wild-type (3.94 ± 1.28 μm versus 3.05 ± 0.95 μm for *pom1*Δ and wild-type, respectively; n = 40 cells; p = 0.000196; two-tailed t test) and displaced from the cellular equator toward a non-growing cell tip (Figure 3B; n = 27 out of 34 cells). Cortical myosin nodes exhibited mobility profiles comparable to control (Figure S3B). Mid1-GFP, Cdr2-GFP, and mCherry-Rng2 also relocalized away from cell equator (Figures 3B and S3C). Cdc15-GFP was found in a sock-like pattern at non-growing tips of interphase *pom1*Δ cells (Figure 3B). This was surprising because, in *S. pombe*, Cdc15 partitions to the cortical actin-rich domains active in endocytosis [27, 28]. Indeed, Cdc15 was enriched at the growing tip in *pom1*Δ *S. pombe* cells (Figure S3D).

During division of *pom1*Δ cells, the off-center actomyosin rings assembled slower than in the wild-type (18.3 ± 4.3 min versus 11.0 ± 1.3 min for *pom1*Δ [n = 14 cells] and the control [n = 12 cells], respectively; p = 1.73E−05; two-tailed t test). Ring constriction also occurred at a decreased rate (25.4 ± 5.5 min; n = 7 cells; p = 0.0208; two-tailed t test). Ring misplacement lead to the birth of two unequally sized daughter cells (Figures 3C and S3E; 11 out of 14 cells divided asymmetrically).

Similarly to single *pom1*Δ cells, double-mutant *pom1*Δ*mid1*Δ *S. japonicus* also mispositioned the division site (Figures 3D and S3F; 9 out of 11 cells divided asymmetrically). Rings formed at the boundary of Cdc15-rich cortical domain at a non-growing tip of *pom1*Δ*mid1*Δ cells (Figure 3E). Suggesting that actin cytoskeleton-associated processes controlled Cdc15 localization together with Pom1, Cdc15-GFP localized throughout the cellular cortex in *pom1*Δ *S. japonicus* cells treated with the actin polymerization inhibitor latrunculin A (Figure S3G). We concluded that, in *S. japonicas*, Pom1 plays a role in positioning the actomyosin ring through regulating the subcellular distribution of the cortical anchor Cdc15.

In *S. pombe*, the centrally positioned nucleus promotes equatorial ring assembly early in mitosis by exporting nuclear Mid1 to the adjacent cortex [29]. We wondered whether replacing the *mid1* gene in *S. japonicus* with its *S. pombe* ortholog could re-establish the functional link between the nucleus and division site selection. The GFP-tagged Mid1$^{S.pombe}$ (GFP-Mid1$^{S.p.}$) knocked in into the native chromosomal locus localized to the nucleus in interphase *S. japonicus*. It redistributed to the lateral cortex early in mitosis (Figure 4A; n = 9 cells). Following the mitotic exit, GFP-Mid1$^{S.p.}$ was incorporated into the actomyosin ring (Figure S4A; 43 out of 50 cells; see also Figure S4B). The timing of ring assembly (13.0 ± 3.0 min; n = 8 cells) and ring constriction (20.2 ± 4.1 min; n = 11 cells) in GFP-Mid1$^{S.p.}$cells was comparable to the wild-type (p = 0.113 and 0.410, respectively; two-tailed t test). The nucleus-derived Mid1$^{S.p}$ was effective in instructing an off-center actomyosin assembly in mitotic *S. japonicus* when nuclei were displaced toward cell tips by centrifugation (Figures S4C and S4D). We observed similar phenotype in cells where *S. japonicus* Mid1 was fused to the SV40-derived NLS (Figure S4D).

We then asked whether GFP-Mid1$^{S.p.}$ could rescue the division-site-positioning defect associated with the lack of Pom1. Indeed, GFP-Mid1$^{S.p.}$*pom1*Δ cells frequently assembled centrally positioned actomyosin rings (Figure 4B; n = 27 out of 42 cells). As expected from the exclusively nuclear localization of GFP-Mid1$^{S.p.}$ during interphase, the myosin complex was not recruited to the medial cortex of GFP-Mid1$^{S.p.}$ cells at this cell-cycle stage (Figure 4C; first time point). However, following Mid1$^{S.p.}$ export from the nucleus, Rlc1-mCherry-marked myosin II colocalized with GFP-Mid1$^{S.p.}$ at the cellular equator (Figure 4C; n = 11 out of 12 cells; see also Figure S4B). Thus, it appeared that Mid1$^{S.p.}$ promoted medial actomyosin assembly in *S. japonicus*, possibly through providing early cortical anchor points for the myosin II and alleviating the Cdc15 cortical patterning defect associated with the loss of Pom1 function. Replacement of the *S. pombe* Mid1 with its *S. japonicus* counterpart virtually phenocopied *mid1* deletion (Figure S4E).

Shifting the anaphase nuclei away from cell center in GFP-Mid1$^{S.p.}$*pom1*Δ cells led to two major functional outcomes. If the nucleus was displaced toward the non-growing tip, efflux of Mid1$^{S.p.}$ from the nucleus early in mitosis promoted an off-center actomyosin ring assembly (Figure 4D; n = 12 cells). However, if the nucleus was shifted toward the growing tip, myosin failed to organize into a ring overlying the nucleus and instead formed long cable-like structures resulting in assembly of tilted and long axis septa (Figure S4F; six out of eight cells). Thus, polarized growth machinery appeared refractory to actomyosin ring anchorage, presumably through conflicted cortical patterning of the myosin II anchors Mid1 and Cdc15 in GFP-Mid1$^{S.p.}$*pom1*Δ cells. Our results suggested that replacement of *S. japonicus* Mid1 with its *S. pombe* ortholog was sufficient to reconstruct the nucleus-instructive mode of ring assembly at the cellular equator.

Genomes of all fission yeasts encode two anillin-like proteins, orthologs of *S. pombe* Mid1 and Mid2 that in turn exhibit similarity to each other in the C-terminally located anillin homology domain and the PH-domain. Phylogenetic tree analysis of the fungal anillins revealed that genomes of every species with the exception of fission yeasts carried one anillin homolog clustering with Mid2 (Figure S4G). The genomes of other Taphrinomycotina [30] also carried a single *mid2*-like gene, suggesting that the Mid1-Mid2 pair could have arisen by duplication of a Mid2/Bud4-like protein in the ancestor of fission yeasts. Divergent

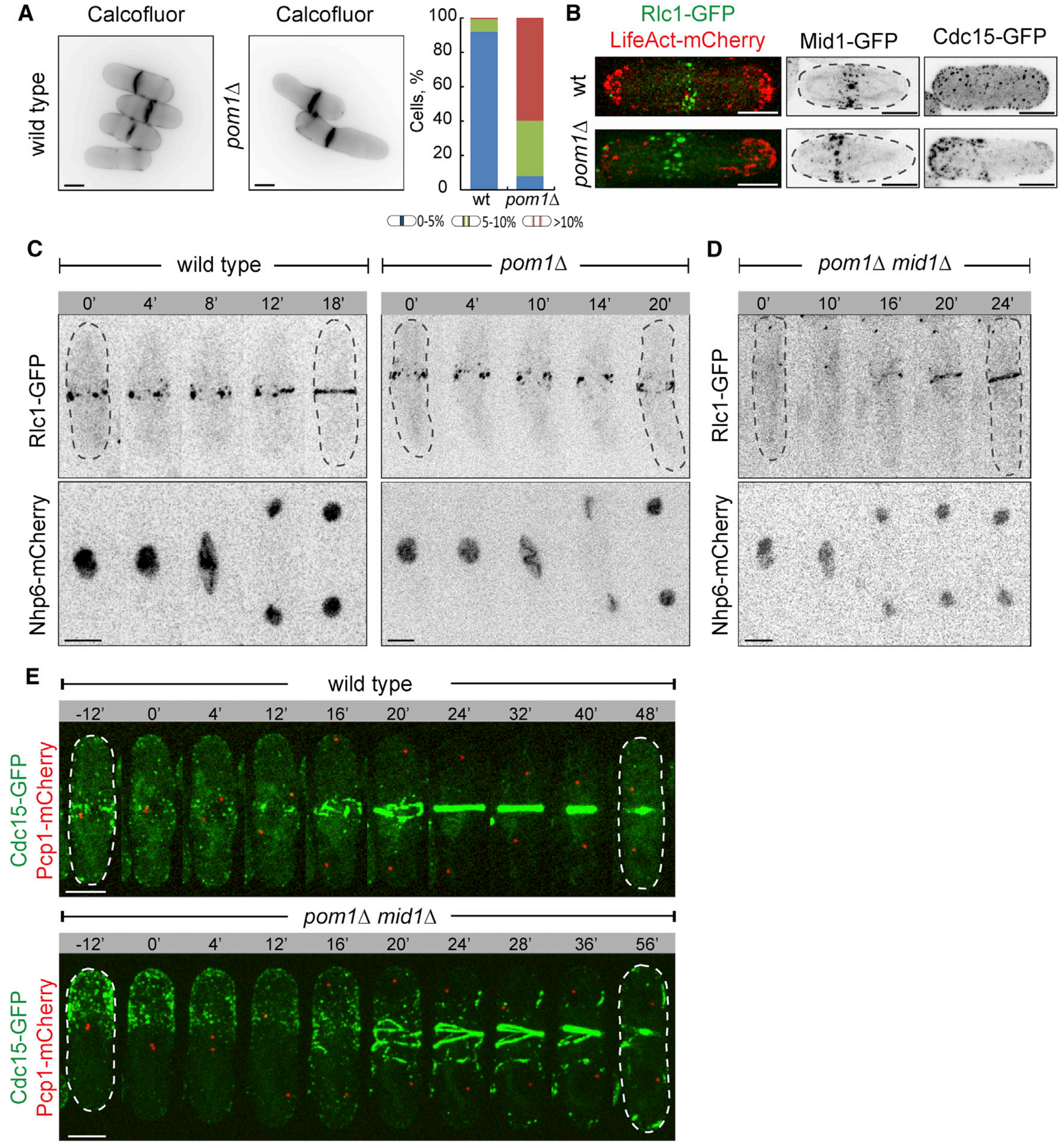

Figure 3. *S. japonicus* Cells Lacking the Polarisome Kinase Pom1 Misposition the Division Site

(A) Calcofluor staining of wild-type and *pom1*Δ *S. japonicus* cells. Plot (right) shows proportion of cells exhibiting division septa at various positions along the long cell axis. 150 cells were counted for each genotype.

(B) Maximum-projection images of interphase wild-type (top) and *pom1*Δ (bottom) *S. japonicus* cells expressing Rlc1-GFP and LifeAct-mCherry, Mid1-GFP, and Cdc15-GFP. The monopolar growth pattern in *pom1*Δ cells is revealed by LifeAct-mCherry.

(C) Time-lapse maximum-projection images of wild-type (left) and *pom1*Δ (right) *S. japonicus* cells co-expressing Rlc1-GFP and Nhp6-mCherry.

(D) A montage of time-lapse maximum-projection images of *pom1*Δ*mid1*Δ *S. japonicus* cells co-expressing Rlc1-GFP and Nhp6-mCherry.

(E) Time-lapse maximum-projection images of wild-type (top) and *pom1*Δ*mid1*Δ (bottom) *S. japonicus* cells co-expressing Cdc15-GFP and Pcp1-mCherry (n = 6 cells). Dashed lines indicate cell boundaries.

For (C)–(E), time is in minutes. Scale bars represent 5 μm. See also Figure S3.

regulation of gene expression in the duplicated pair may have driven early subfunctionalization (Figure S1F) [14–16, 31], with *S. pombe* eventually utilizing Mid1 for nucleus-dependent division site placement.

An evolutionarily conserved function for anillins appears to be scaffolding the cellular division machinery including actomyosin and septins [32–38]. Mid1 plays such structural role in *S. japonicus*, tethering the myosin II to the equatorial cortex in interphase and promoting recruitment of the mitotic anchor Cdc15 early in mitosis (Figures 1C, 2B, S2C, and S2D). Although the equatorial band of myosin is dispensable for medial division, it may enhance the fidelity of division site

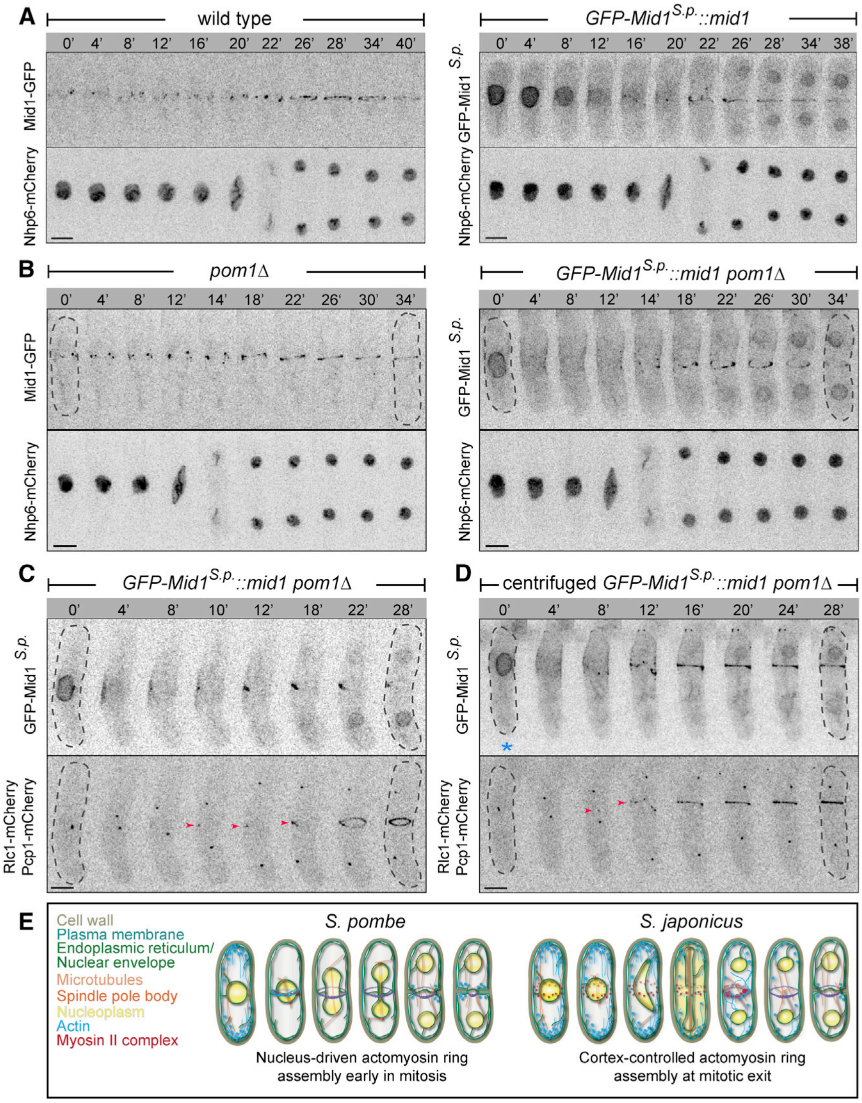

Figure 4. Replacement of *S. japonicus* Mid1 with Its *S. pombe* Ortholog Is Sufficient to Reconstruct Nucleus-Driven Division Site Positioning

(A) Time-lapse maximum-projection images of *S. japonicus* cells co-expressing the endogenous GFP-tagged Mid1 (left) or its *S. pombe* ortholog GFP-Mid1$^{S.p.}$ (right) together with the nuclear marker Nhp6-mCherry.

(B) Time-lapse maximum-projection images of *pom1Δ S. japonicus* cells co-expressing either Mid1-GFP (left) or GFP-Mid1$^{S.p.}$ (right) and Nhp6-mCherry.

(C) Time-lapse maximum-projection images of *pom1Δ S. japonicus* cells co-expressing GFP-Mid1$^{S.p.}$ with Rlc1-mCherry and the SPB marker Pcp1-mCherry to indicate mitotic progression. Dashed lines indicate cell boundaries. Red arrowheads indicate Rlc1-mCherry signal at the equatorial cortex.

(D) Time-lapse maximum-projection images of *pom1Δ S. japonicus* cell co-expressing GFP-Mid1$^{S.p.}$, Rlc1-mCherry and Pcp1-mCherry, where the nucleus was displaced toward a non-growing end by centrifugation. Dashed lines indicate cell boundaries. Red arrowheads indicate Rlc1-mCherry signal at the cortex. Blue asterisk indicates a growing cell tip.

(E) Diagrams summarizing the modes of cytokinesis in *S. pombe* and *S. japonicus*.

For (A)–(D), time is in minutes. Scale bars represent 5 μm. See also Figure S4.

positioning upon insults to intracellular organization (Figures 1F and 1G).

As *S. japonicus* cells enter mitosis, the F-BAR protein Cdc15 takes over from Mid1 as a primary cortical tether for the actomyosin (Figure 2A). The key difference from *S. pombe* is that Cdc15 is able to localize to the cellular equator in the absence of Mid1 by utilizing signaling cues originating at the cell tips. Pom1 kinase and the actin-dependent growth machinery appear to function in specifying cortical distribution of Cdc15 (Figures 3 and S3G). Using cortical cues to control actomyosin tethering may allow *S. japonicus* to dynamically position the division site by reading out the state of the polarized growth machinery, rather than through tracking the nucleus. Unlike *S. pombe*, *S. japonicus* yeast cells readily transit to hyphal growth [39] and it would be of interest to determine whether controlling division by cortical signals provides physiological advantage in the context of a multicellular colony.

Unlike many cell types including *S. japonicus* and mammalian cells [1], *S. pombe* assembles the division rings early in mitosis [40]. Although the cortical actomyosin cables can be generated in *S. pombe mid1Δ* cells, they do not compact into orthogonal rings. Arguing for the lack of temporal coordination between actomyosin contractility and activation of septum assembly, inhibition of septum deposition allows *mid1Δ* cells sufficient time to correct ring orientation [11]. An interesting possibility is that precocious Mid1-dependent ring assembly may have arisen in *S. pombe* lineage to compensate for deregulation of the conventional, late mitotic pathway. An unrelated nuclear function in regulation of gene expression [41] could have promoted evolutionary co-option of Mid1 in instructive division site positioning in addition to its structural role in scaffolding cell division machinery.

The mitotic nucleus appears to influence ring assembly in *S. japonicus* by promoting actin polymerization. Possible signals could include local release of an activator of actin polymerization following NE breakdown or physical proximity to regulators localizing to the spindle or the SPBs.

Our work outlines a set of rules for generating functional diversity in division site placement (Figure 4E). Importantly, NE breakdown and post-mitotic assembly of a medial actomyosin ring position *S. japonicus* as a valuable genetically tractable model for understanding the logic of cytokinesis regulation used by higher eukaryotes.

EXPERIMENTAL PROCEDURES

Yeast Strains and Culture Conditions

S. japonicus and *S. pombe* strains used in this study and their genotypes are listed in Table S1. *S. pombe* growth media and genetic methods were according to [42]. Auxotrophic heterothallic "wild-type" *S. japonicus* strains were a kind gift from H. Niki [43]. *S. japonicus* culture conditions and DNA transformation were as described in [44]. For details of strain construction, gene-expression analysis, and imaging methods, see the Supplemental Experimental Procedures.

SUPPLEMENTAL INFORMATION

Supplemental Information includes Supplemental Experimental Procedures, four figures, one table, and three movies and can be found with this article online at http://dx.doi.org/10.1016/j.cub.2015.02.056.

ACKNOWLEDGMENTS

We are grateful to M. Balasubramanian and E. Makeyev for suggestions on the manuscript, the Nikon Imaging Centre at King's College London for help with confocal microscopy, and S.O. lab members for discussions throughout this work. We would like to thank A. Vjestica for assistance with artwork, H. Niki for auxotrophic heterothallic strains of *Schizosaccharomyces japonicus*, and J. Stajich and G. Jedd for the access to Neolecta genome sequence. Our work was supported by King's College London and the Wellcome Trust Senior Investigator Award (103741/Z/14/Z) to S.O.

Received: July 25, 2014
Revised: January 29, 2015
Accepted: February 19, 2015
Published: April 9, 2015

REFERENCES

1. Oliferenko, S., Chew, T.G., and Balasubramanian, M.K. (2009). Positioning cytokinesis. Genes Dev. *23*, 660–674.
2. Lee, I.J., Coffman, V.C., and Wu, J.Q. (2012). Contractile-ring assembly in fission yeast cytokinesis: Recent advances and new perspectives. Cytoskeleton (Hoboken) *69*, 751–763.
3. Alfa, C.E., and Hyams, J.S. (1990). Distribution of tubulin and actin through the cell division cycle of the fission yeast Schizosaccharomyces japonicus var. versatilis: a comparison with Schizosaccharomyces pombe. J. Cell Sci. *96*, 71–77.
4. Aoki, K., Hayashi, H., Furuya, K., Sato, M., Takagi, T., Osumi, M., Kimura, A., and Niki, H. (2011). Breakage of the nuclear envelope by an extending mitotic nucleus occurs during anaphase in Schizosaccharomyces japonicus. Genes Cells *16*, 911–926.
5. Yam, C., He, Y., Zhang, D., Chiam, K.H., and Oliferenko, S. (2011). Divergent strategies for controlling the nuclear membrane satisfy geometric constraints during nuclear division. Curr. Biol. *21*, 1314–1319.
6. Motegi, F., Mishra, M., Balasubramanian, M.K., and Mabuchi, I. (2004). Myosin-II reorganization during mitosis is controlled temporally by its dephosphorylation and spatially by Mid1 in fission yeast. J. Cell Biol. *165*, 685–695.
7. Chang, F., Woollard, A., and Nurse, P. (1996). Isolation and characterization of fission yeast mutants defective in the assembly and placement of the contractile actin ring. J. Cell Sci. *109*, 131–142.
8. Bähler, J., Steever, A.B., Wheatley, S., Wang, Yl., Pringle, J.R., Gould, K.L., and McCollum, D. (1998). Role of polo kinase and Mid1p in determining the site of cell division in fission yeast. J. Cell Biol. *143*, 1603–1616.
9. Sohrmann, M., Fankhauser, C., Brodbeck, C., and Simanis, V. (1996). The dmf1/mid1 gene is essential for correct positioning of the division septum in fission yeast. Genes Dev. *10*, 2707–2719.
10. Hachet, O., and Simanis, V. (2008). Mid1p/anillin and the septation initiation network orchestrate contractile ring assembly for cytokinesis. Genes Dev. *22*, 3205–3216.
11. Huang, Y., Yan, H., and Balasubramanian, M.K. (2008). Assembly of normal actomyosin rings in the absence of Mid1p and cortical nodes in fission yeast. J. Cell Biol. *183*, 979–988.
12. Tasto, J.J., Morrell, J.L., and Gould, K.L. (2003). An anillin homologue, Mid2p, acts during fission yeast cytokinesis to organize the septin ring and promote cell separation. J. Cell Biol. *160*, 1093–1103.
13. Berlin, A., Paoletti, A., and Chang, F. (2003). Mid2p stabilizes septin rings during cytokinesis in fission yeast. J. Cell Biol. *160*, 1083–1092.
14. Rustici, G., Mata, J., Kivinen, K., Lió, P., Penkett, C.J., Burns, G., Hayles, J., Brazma, A., Nurse, P., and Bähler, J. (2004). Periodic gene expression program of the fission yeast cell cycle. Nat. Genet. *36*, 809–817.
15. Oliva, A., Rosebrock, A., Ferrezuelo, F., Pyne, S., Chen, H., Skiena, S., Futcher, B., and Leatherwood, J. (2005). The cell cycle-regulated genes of Schizosaccharomyces pombe. PLoS Biol. *3*, e225.
16. Peng, X., Karuturi, R.K., Miller, L.D., Lin, K., Jia, Y., Kondu, P., Wang, L., Wong, L.S., Liu, E.T., Balasubramanian, M.K., and Liu, J. (2005). Identification of cell cycle-regulated genes in fission yeast. Mol. Biol. Cell *16*, 1026–1042.
17. Paoletti, A., and Chang, F. (2000). Analysis of mid1p, a protein required for placement of the cell division site, reveals a link between the nucleus and the cell surface in fission yeast. Mol. Biol. Cell *11*, 2757–2773.
18. Celton-Morizur, S., Bordes, N., Fraisier, V., Tran, P.T., and Paoletti, A. (2004). C-terminal anchoring of mid1p to membranes stabilizes cytokinetic ring position in early mitosis in fission yeast. Mol. Cell. Biol. *24*, 10621–10635.
19. Kalderon, D., Roberts, B.L., Richardson, W.D., and Smith, A.E. (1984). A short amino acid sequence able to specify nuclear location. Cell *39*, 499–509.
20. Tran, P.T., Marsh, L., Doye, V., Inoué, S., and Chang, F. (2001). A mechanism for nuclear positioning in fission yeast based on microtubule pushing. J. Cell Biol. *153*, 397–411.
21. Roberts-Galbraith, R.H., Chen, J.S., Wang, J., and Gould, K.L. (2009). The SH3 domains of two PCH family members cooperate in assembly of the Schizosaccharomyces pombe contractile ring. J. Cell Biol. *184*, 113–127.
22. Roberts-Galbraith, R.H., Ohi, M.D., Ballif, B.A., Chen, J.S., McLeod, I., McDonald, W.H., Gygi, S.P., Yates, J.R., 3rd, and Gould, K.L. (2010). Dephosphorylation of F-BAR protein Cdc15 modulates its conformation and stimulates its scaffolding activity at the cell division site. Mol. Cell *39*, 86–99.
23. Laporte, D., Coffman, V.C., Lee, I.J., and Wu, J.Q. (2011). Assembly and architecture of precursor nodes during fission yeast cytokinesis. J. Cell Biol. *192*, 1005–1021.
24. Huang, Y., Chew, T.G., Ge, W., and Balasubramanian, M.K. (2007). Polarity determinants Tea1p, Tea4p, and Pom1p inhibit division-septum assembly at cell ends in fission yeast. Dev. Cell *12*, 987–996.
25. Celton-Morizur, S., Racine, V., Sibarita, J.B., and Paoletti, A. (2006). Pom1 kinase links division plane position to cell polarity by regulating Mid1p cortical distribution. J. Cell Sci. *119*, 4710–4718.
26. Padte, N.N., Martin, S.G., Howard, M., and Chang, F. (2006). The cell-end factor pom1p inhibits mid1p in specification of the cell division plane in fission yeast. Curr. Biol. *16*, 2480–2487.
27. Carnahan, R.H., and Gould, K.L. (2003). The PCH family protein, Cdc15p, recruits two F-actin nucleation pathways to coordinate cytokinetic actin ring formation in Schizosaccharomyces pombe. J. Cell Biol. *162*, 851–862.
28. Arasada, R., and Pollard, T.D. (2011). Distinct roles for F-BAR proteins Cdc15p and Bzz1p in actin polymerization at sites of endocytosis in fission yeast. Curr. Biol. *21*, 1450–1459.
29. Rincon, S.A., and Paoletti, A. (2012). Mid1/anillin and the spatial regulation of cytokinesis in fission yeast. Cytoskeleton (Hoboken) *69*, 764–777.
30. Schoch, C.L., Sung, G.H., López-Giráldez, F., Townsend, J.P., Miadlikowska, J., Hofstetter, V., Robbertse, B., Matheny, P.B., Kauff, F.,

Wang, Z., et al. (2009). The Ascomycota tree of life: a phylum-wide phylogeny clarifies the origin and evolution of fundamental reproductive and ecological traits. Syst. Biol. *58*, 224–239.

31. Force, A., Lynch, M., Pickett, F.B., Amores, A., Yan, Y.L., and Postlethwait, J. (1999). Preservation of duplicate genes by complementary, degenerative mutations. Genetics *151*, 1531–1545.
32. Field, C.M., and Alberts, B.M. (1995). Anillin, a contractile ring protein that cycles from the nucleus to the cell cortex. J. Cell Biol. *131*, 165–178.
33. Oegema, K., Savoian, M.S., Mitchison, T.J., and Field, C.M. (2000). Functional analysis of a human homologue of the Drosophila actin binding protein anillin suggests a role in cytokinesis. J. Cell Biol. *150*, 539–552.
34. Straight, A.F., Field, C.M., and Mitchison, T.J. (2005). Anillin binds nonmuscle myosin II and regulates the contractile ring. Mol. Biol. Cell *16*, 193–201.
35. Piekny, A.J., and Glotzer, M. (2008). Anillin is a scaffold protein that links RhoA, actin, and myosin during cytokinesis. Curr. Biol. *18*, 30–36.
36. Si, H., Rittenour, W.R., Xu, K., Nicksarlian, M., Calvo, A.M., and Harris, S.D. (2012). Morphogenetic and developmental functions of the Aspergillus nidulans homologues of the yeast bud site selection proteins Bud4 and Axl2. Mol. Microbiol. *85*, 252–270.
37. Sanders, S.L., and Herskowitz, I. (1996). The BUD4 protein of yeast, required for axial budding, is localized to the mother/BUD neck in a cell cycle-dependent manner. J. Cell Biol. *134*, 413–427.
38. Eluère, R., Varlet, I., Bernadac, A., and Simon, M.N. (2012). Cdk and the anillin homolog Bud4 define a new pathway regulating septin organization in yeast. Cell cycle *11*, 151–158.
39. Furuya, K., and Niki, H. (2010). The DNA damage checkpoint regulates a transition between yeast and hyphal growth in Schizosaccharomyces japonicus. Mol. Cell. Biol. *30*, 2909–2917.
40. Guertin, D.A., Chang, L., Irshad, F., Gould, K.L., and McCollum, D. (2000). The role of the sid1p kinase and cdc14p in regulating the onset of cytokinesis in fission yeast. EMBO J. *19*, 1803–1815.
41. Agarwal, M., Papadopoulou, K., Mayeux, A., Vajrala, V., Quintana, D.M., Paoletti, A., and McInerny, C.J. (2010). Mid1p-dependent regulation of the M-G1 transcription wave in fission yeast. J. Cell Sci. *123*, 4366–4373.
42. Gould, K.L. (2004). Protocols for experimentation with Schizosaccharomyces pombe. Methods *33*, 187–188.
43. Furuya, K., and Niki, H. (2009). Isolation of heterothallic haploid and auxotrophic mutants of Schizosaccharomyces japonicus. Yeast *26*, 221–233.
44. Aoki, K., Nakajima, R., Furuya, K., and Niki, H. (2010). Novel episomal vectors and a highly efficient transformation procedure for the fission yeast Schizosaccharomyces japonicus. Yeast *27*, 1049–1060.

Current Biology
Supplemental Information

Rewiring of Cellular Division Site Selection
in Evolution of Fission Yeasts

Ying Gu, Candice Yam, and Snezhana Oliferenko

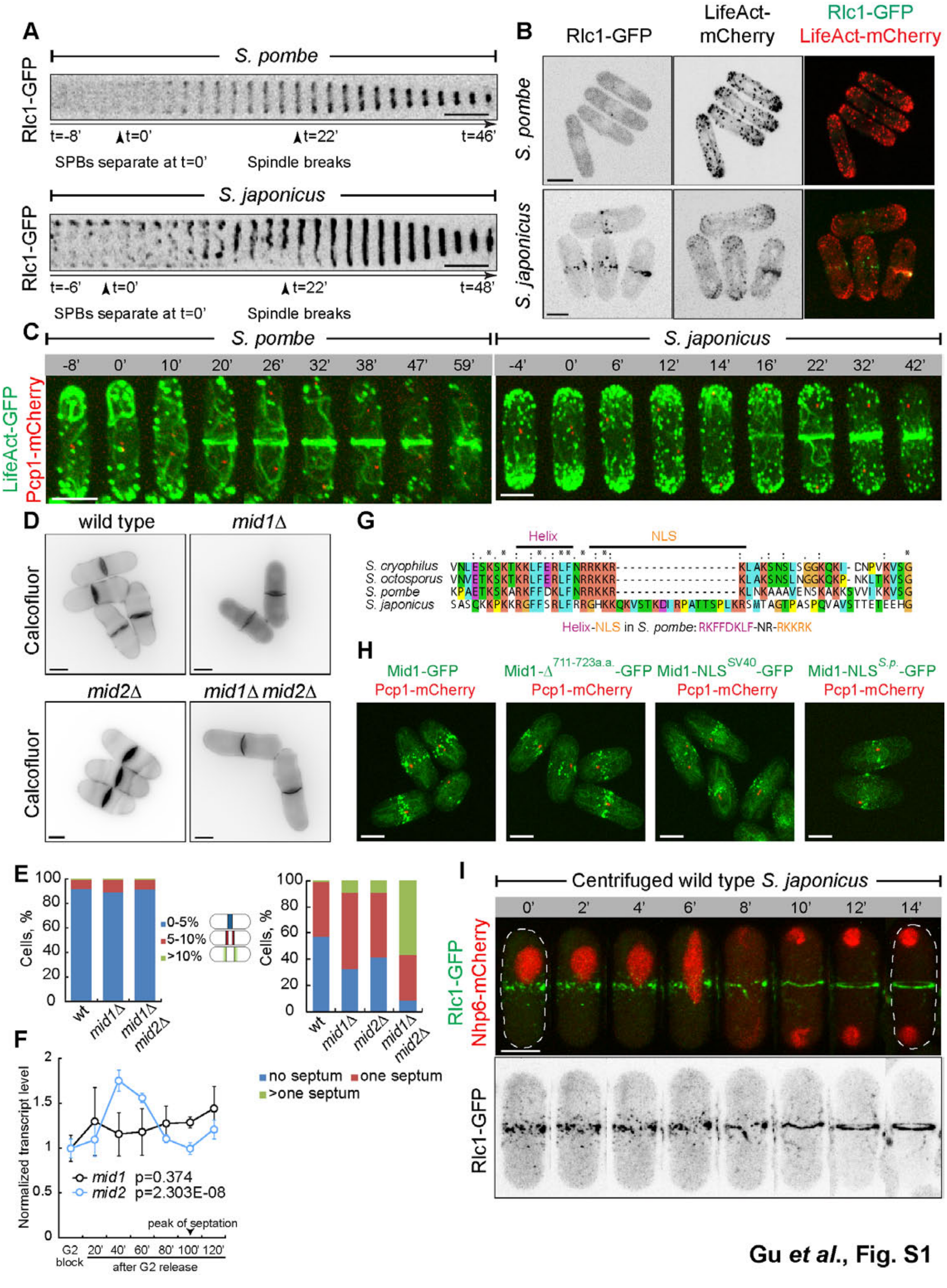

Gu *et al.*, Fig. S1

Figure S1, related to Figure 1

(**A**) Time-lapse maximum-projection images of Rlc1-GFP dynamics at the equatorial region of dividing *S. pombe* (top) and *S. japonicus* (bottom) cells. Mitotic progression has been monitored using Pcp1-mCherry-marked SPBs (see timeline underneath each montage). Time interval is 2 minutes. Arrowheads indicate reference time points. (**B**) Maximum-projection images of *S. pombe* (top) and *S. japonicus* (bottom) cells co-expressing Rlc1-GFP and the F-actin marker LifeAct-mCherry. Presented are individual channels and color composite images. The myosin II complex localizes to the medial cortex of interphase *S. japonicus* but not *S. pombe* cells. (**C**) Color composites of time-lapse maximum-projection images of *S. pombe* (left) and *S. japonicus* (right) cells co-expressing LifeAct-GFP and the SPB marker Pcp1-mCherry. n=13 cells for *S. pombe* and 25 cells for *S. japonicus*. (**D**) Calcofluor staining of wild type, *mid1*Δ, *mid2*Δ and *mid1*Δ*mid2*Δ *S. japonicus* cells. (**E**) Graph (left) showing proportion of cells exhibiting division septa at various positions along the long cell axis. Graph (right) showing fractions of cells with septa in asynchronously growing cell populations of indicated genotypes. We counted 151 wild type, 213 *mid1*Δ, 365 *mid2*Δ and 166 *mid1*Δ*mid2*Δ cells. (**F**) Normalized transcript levels of *mid1* and *mid2* at indicated time points in *cdc25-D9* block and release experiment. Error bars represent standard deviation. p-value estimated by ANOVA (one-way) test. (**G**) Sequence alignment of Mid1 proteins at the Helix-NLS region within the *Schizosaccharomyces* clade. (**H**) Color composites of maximum-projection images of *S. japonicus* cells co-expressing Mid1-GFP or indicated GFP-tagged Mid1 variants with the SPB marker Pcp1-mCherry. Mid1-GFP expressing cells, n=31; Mid1-$\Delta^{711\text{-}723\text{a.a.}}$-GFP cells, n=25. 83.3% of interphase Mid1-NLS^{SV40}-GFP cells (n=54) and 60% of interphase Mid1-NLS$^{S.p.}$-GFP cells (n=35) showed nuclear enrichment of the Mid1 mutant proteins. (**I**) Time-lapse maximum-projection images of a centrifuged *S. japonicus* cell co-expressing Rlc1-GFP and the nucleoplasm marker Nhp6-mCherry shown as the color composite (top) and the individual GFP channel (bottom). Dashed lines indicate cell boundaries. (**A**, **C**, **I**) Time is in minutes. Scale bars, 5 μm.

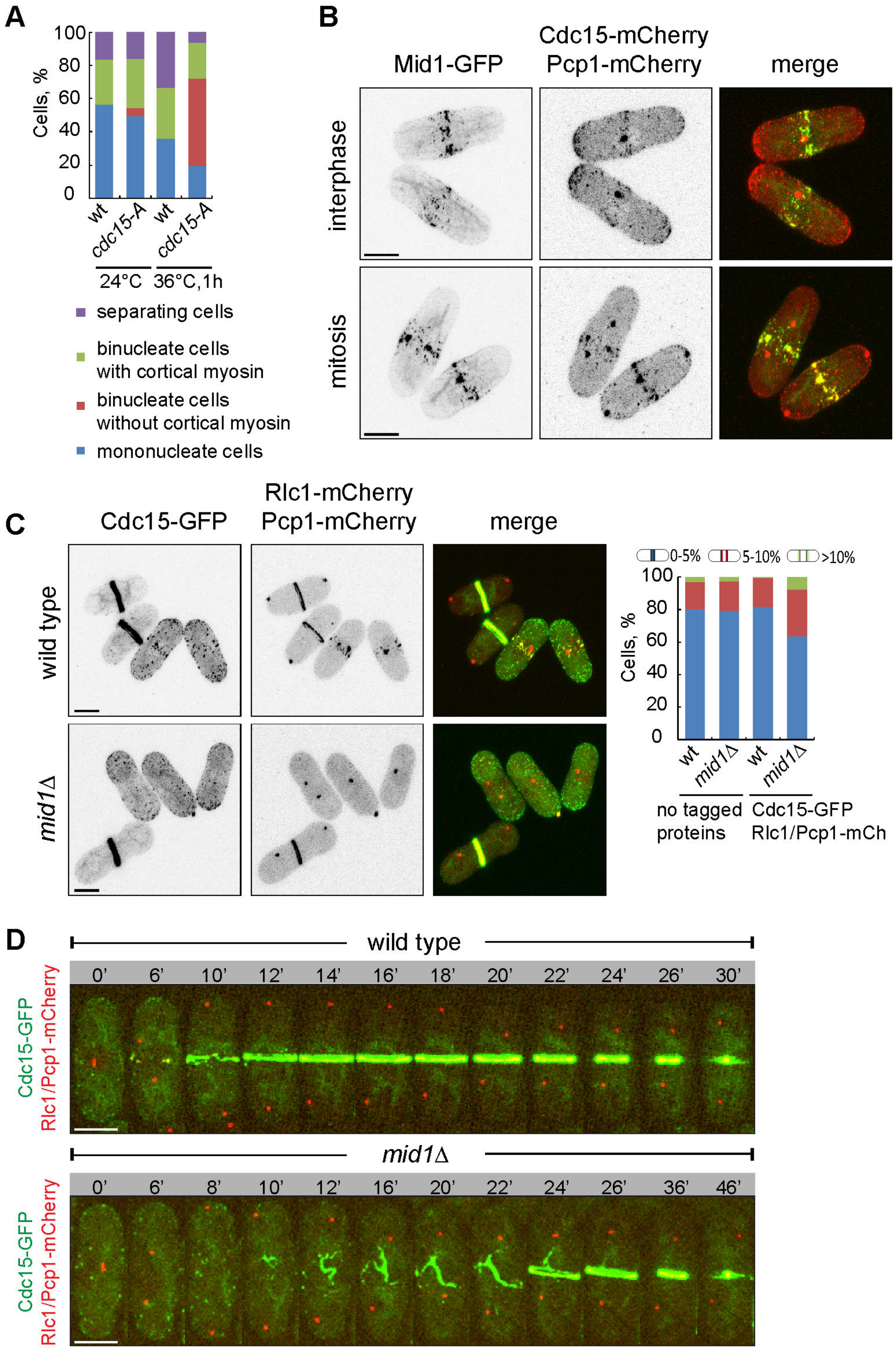

Gu *et al.*, Fig. S2

Figure S2, related to Figure 2

(**A**) Graph representing fractions of Rlc1-GFP-expressing cells exhibiting cell division phenotypes at the permissive temperature of 24°C or following incubation at the restrictive temperature of 36°C for 1 hour. Phenotypes were quantified in asynchronously growing cell populations. (**B**) Maximum-projection images of interphase and mitotic wild type *S. japonicus* cells co-expressing Mid1-GFP, Cdc15-mCherry and Pcp1-mCherry. Shown are individual channels and the composite color images. (**C**) Maximum-projection images of wild type (top) and *mid1*Δ (bottom) *S. japonicus* cells co-expressing Cdc15-GFP, Rlc1-mCherry and Pcp1-mCherry. Shown are individual channels and the composite color images. Of note, simultaneous presence of fluorophore-tagged Cdc15 and Rlc1 results in mild actomyosin ring positioning defects shown in the graph at far right. The graph represents fractions of Calcofluor-stained cells with septa at various cellular positions in asynchronously growing populations of indicated genotypes at 30°C. Wild type with no tagged proteins, n=210 cells; *mid1*Δ with no tagged proteins, n=195 cells; wild type with tagged proteins, n=206 cells; *mid1*Δ with tagged proteins, n=189 cells. (**D**) Color composites of time-lapse maximum-projection images of wild type (top) and *mid1*Δ (bottom) *S. japonicus* cells co-expressing Cdc15-GFP, Rlc1-mCherry and Pcp1-mCherry. Time is in minutes. Scale bars, 5 μm.

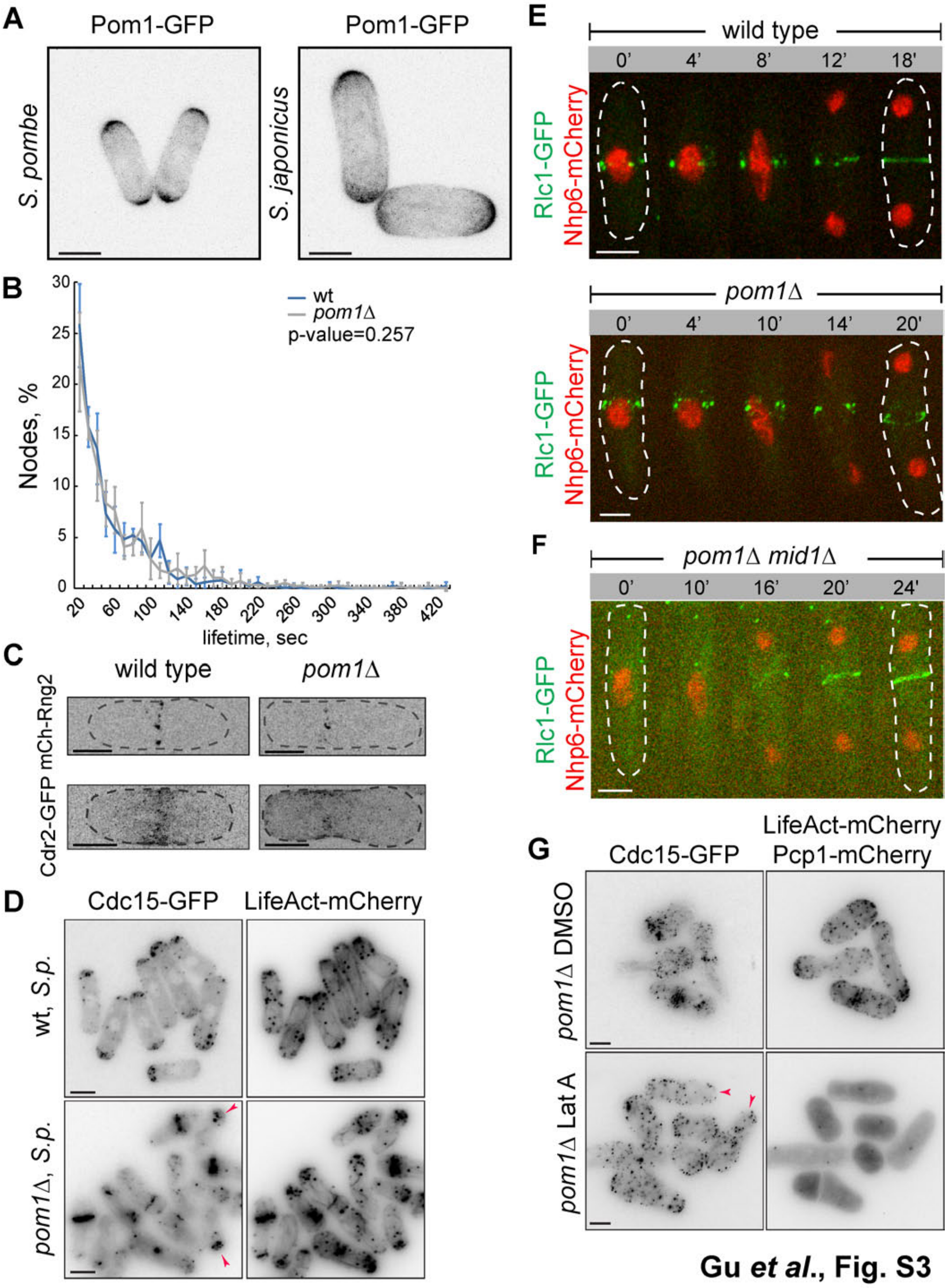

Gu *et al.*, Fig. S3

Figure S3, related to Figure 3

(**A**) Maximum-projection images of *S. pombe* (left) and *S. japonicus* (right) cells expressing Pom1-GFP. (**B**) Distribution of mobility profiles of interphase myosin nodes in wild type and *pom1Δ* cells, plotting the percentages of myosin nodes (Rlc1-GFP) with indicated lifetimes (dwelling times) at the medial cortex. n=5 cells. Error bars represent standard deviation. p-value estimated by two-sample Kolmogorov-Smirnov test. (**C**) Maximum-projection images of interphase wild type and *pom1*Δ *S. japonicus* cells expressing mCherry-Rng2 or Cdr2-GFP. (**D**) Maximum-projection images of wild type (top) and *pom1*Δ (bottom) *S. pombe* cells co-expressing Cdc15-GFP and LifeAct-mCherry. Shown are individual channels. Red arrowheads indicate the growing cell tip marked by LifeAct-mCherry. *S. p.*, *S. pombe*. (**E**) Color composites of time-lapse maximum-projection images of wild type (top) and *pom1*Δ (bottom) *S. japonicus* cells co-expressing Rlc1-GFP and Nhp6-mCherry, presented in Fig. 3C. (**F**) Color composites of time-lapse maximum-projection images of *pom1*Δ*mid1*Δ *S. japonicus* cell co-expressing Rlc1-GFP and Nhp6-mCherry, presented in Fig. 3D. (**G**) Maximum-projection images of *pom1*Δ *S. japonicus* cells co-expressing Cdc15-GFP, LifeAct-mCherry and Pcp1-mCherry treated with DMSO solvent (top) or LatrunculinA (bottom). Shown are individual channel images. (**E** and **F**) Dashed lines indicate cell boundaries. Time is in minutes. Scale bars, 5 μm .

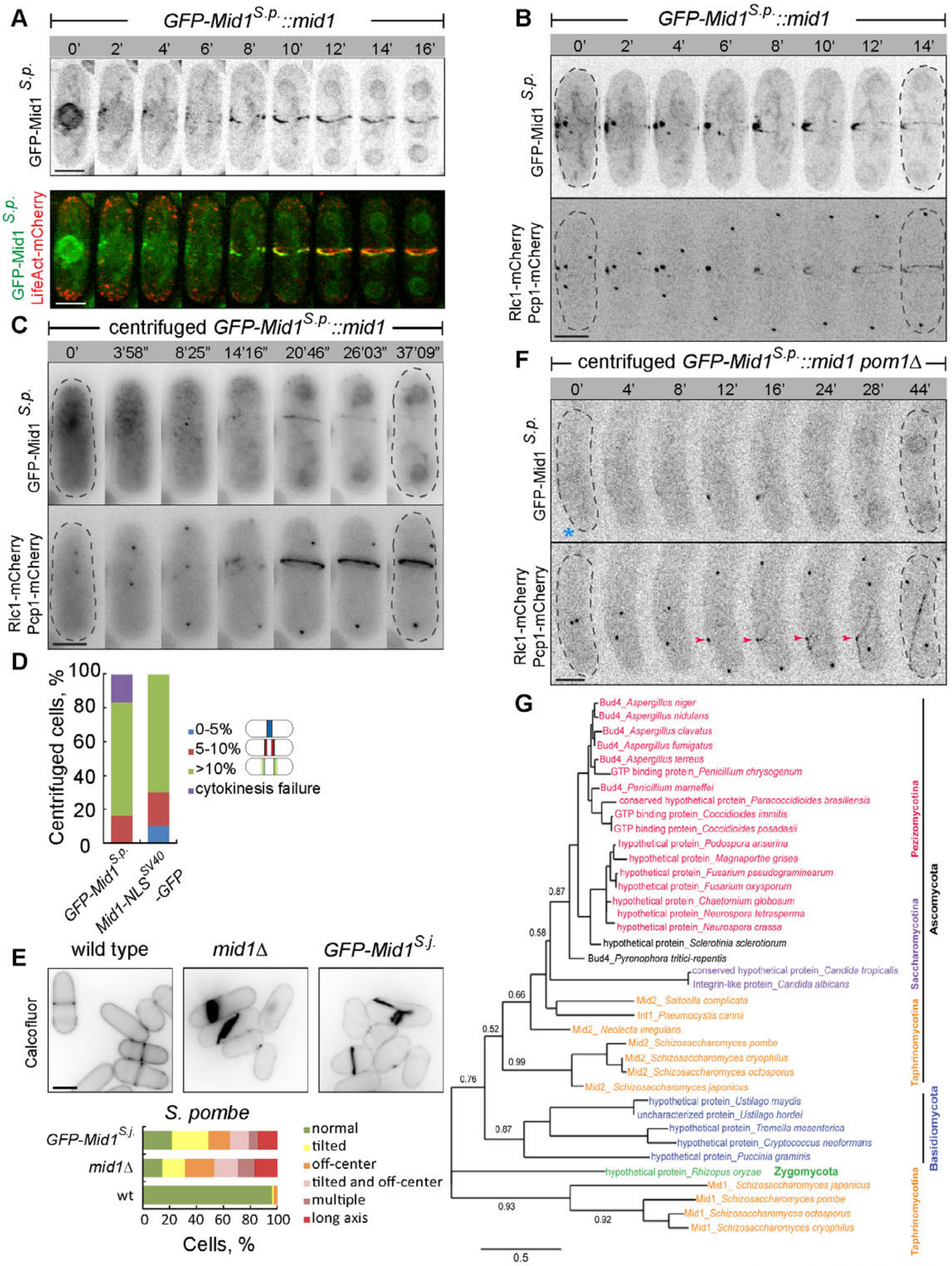

Gu *et al.*, Fig. S4

Figure S4, related to Figure 4

(**A**) Time-lapse maximum-projection images of *S. japonicus* co-expressing GFP-Mid1$^{S.p.}$ and LifeAct-mCherry show that GFP-Mid1$^{S.p.}$ is incorporated into the actomyosin division ring. Shown are the individual GFP channel (top) and the composite color (bottom) images. (**B**) Time-lapse maximum-projection images of *S. japonicus* co-expressing GFP-Mid1$^{S.p.}$ with Rlc1-mCherry and Pcp1-mCherry shows that the myosin II complex colocalizes with GFP-Mid1$^{S.p.}$ at the equatorial cortex early in mitosis. (**C**) Time-lapse single-plane images (upper panel for GFP, bottom panel for mCherry) of *S. japonicus* cell co-expressing GFP-Mid1$^{S.p.}$ with the myosin II light chain Rlc1-mCherry and the SPB marker Pcp1-mCherry, after nuclear displacement by centrifugation. (**D**) Graph representing proportions of GFP-Mid1$^{S.p.}$ and Mid1-NLS^{SV40}-GFP expressing *S. japonicus* cells with septa at indicated cellular positions after centrifugation. GFP-Mid1$^{S.p.}$, n=12 cells; Mid1-NLS^{SV40}-GFP, n=10 cells. (**E**) Calcofluor staining of *S. pombe* cells. Shown are the wild type (left), *mid1*Δ (center) and cells where the endogenous *mid1* has been replaced with its *S. japonicus* ortholog (right). Plot (below) shows proportion of cells exhibiting division septa at various positions along the long cell axis. Wild type cells, n=214; *mid1*Δ cells, n=221; GFP-Mid1$^{S.j.}$ cells, n=263. (**F**) Time-lapse maximum-projection images of *pom1*Δ *S. japonicus* cell co-expressing GFP-Mid1$^{S.p.}$ with the myosin II light chain Rlc1-mCherry and the SPB marker Pcp1-mCherry, where the nucleus was displaced towards a growing end by centrifugation. Note that Rlc1-mCherry fails to assemble into a ring. Red arrowheads indicate Rlc1-mCherry signal at the cortex. Blue asterisk indicates a growing cell tip. For (**A**-**C**, **F**), time is in minutes and seconds. Dashed lines indicate cell boundaries. Scale bars, 5 μm. (**G**) Phylogenetic tree of the anillin-like proteins in the fungal kingdom. A collection of protein sequences were pooled from representative species. Protein sequences were aligned and the phylogenetic tree was generated using Phylogeny (http://www.phylogeny.fr/).

Supplemental Table

Table S1. List of Strains

Schizosaccharomyces japonicus		
**Figure**	**Genotype**	**Collection No.**
1A, 1D, S1A, 2A	*Rlc1-GFP::kan^R Pcp1-mCherry::ura4+::kan^R ura4sj-D3 ade6sj-domE?*	SOJ1445
1B, 1F	*pAct1-Lifeact-GFP::ura4+ Nhp6-mCherry::ura4+ ura4sj-D3 ade6sj-domE?*	SOJ638
1C	*Rlc1-GFP::kan^R ura4sj-D3 h-*	SOJ27
	mid1Δ::ura4+ Rlc1-GFP::kan^R ura4sj-D3 ade6sj-domE?	SOJ1286
	GFP-Myo2::ura4+ura4sj-D3 ade6sj-domE h+	SOJ1556
	mid1Δ::kan^R GFP-Myo2::ura4+ura4sj-D3 ade6sj-domE?	SOJ1558
	GFP-Rng2::ura4 ura4sj-D3 ade6sj-domE h+	SOJ1518
	mid1Δ::kan^R GFP-Rng2::ura4+ ura4sj-D3 ade6sj-domE?	SOJ1539
1D	*mid1Δ::ura4+ Rlc1-GFP::kan^R Pcp1- mCherry::ura4+::kan^R ura4sj-D3 ade6sj-domE?*	SOJ1443
1E, S1H	*Mid1-GFP::ura4+::kan^R Pcp1-mCherry::ura4+::kan^R ura4sj-D3 ade6sj-domE*	SOJ1540
1F	*mid1Δ::ura4+ pAct1-Lifeact-GFP::ura4+ ura4sj-D3 ade6sj-domE*	SOJ2224
S1B, 3B	*Rlc1-GFP::kan^R pAct1-Lifeact-mCherry::ura4+ ura4sj-D3 ade6sj-domE?*	SOJ634
S1C	*pAct1-Lifeact-GFP::ura4+ Pcp1-mCherry::ura4+::kan^R ura4sj-D3 ade6sj-domE*	SOJ1481
S1D, 3A	*ura4sj-D3 ade6sj-domE h+*	SOJ88
S1D	*mid1Δ::ura4+ ura4sj-D3 ade6sj-domE h+*	SOJ680
	mid2Δ::kan^R ura4sj-D3 ade6sj-domE h+	SOJ1158
	mid1Δ::ura4+ mid2Δ::kan^R ura4sj-D3 ade6sj-domE	SOJ1299
S1F	*cdc25-D9::ura4+::kan^R ura4sj-D3 ade6sj-domE*	SOJ732
S1H	*Mid1-Δ$^{711\text{-}723a.a.}$-GFP::ura4+::kan^R Pcp1-mCherry::ura4+::kan^R ura4sj-D3 ade6sj-domE*	SOJ2208
	Mid1-NLS^{SV40}-GFP::ura4+::kan^R Pcp1-mCherry::ura4+::kan^R ura4sj-D3 ade6sj-domE	SOJ2207
	Mid1-NLS$^{S.p.}$-GFP::ura4+ Pcp1-mCherry::ura4+::kan^R ura4sj-D3 ade6sj-domE	SOJ2234
S1I, 3C, S3E	*Rlc1-GFP::kan^R Nhp6-mCherry::ura4+ ura4sj-D3 ade6sj-domE?*	SOJ694
2A	*cdc15-A::ura4+::kan^R Rlc1-GFP::kan^R Pcp1-mCherry::ura4+::kan^R ura4sj-D3 ade6sj-domE?*	SOJ2087
	cdc15-A::ura4+::kan^R mid1Δ::ura4+ Rlc1-GFP::kan^R Pcp1-mCherry::ura4+::kan^R ura4sj-D3 ade6sj-domE?	SOJ2213
2B	*Cdc15-GFP::ura4+::kan^R Rlc1-mCherry::ura4+::kan^R ura4sj-D3 ade6sj-domE*	SOJ2064
	mid1Δ::ura4+ Cdc15-GFP::ura4+::kan^R Rlc1-mCherry::ura4+::kan^R ura4sj-D3 ade6sj-domE	SOJ2062
S2B	*Mid1-GFP::ura4+::kan^R Cdc15-mCherry::ura4+::kan^R Pcp1-mCherry::ura4+::kan^R ura4sj-D3 ade6sj-domE*	SOJ2165
S2C, S2D	*Cdc15-GFP::ura4+::kan^R Rlc1-mCherry::ura4+::kan^R Pcp1-mCherry::ura4+::kan^R ura4sj-D3 ade6sj-domE*	SOJ2069
S2C, S2D	*mid1Δ::ura4+ Cdc15-GFP::ura4+::kan^R Rlc1-mCherry::ura4+::kan^R Pcp1-mCherry::ura4+::kan^R ura4sj-D3 ade6sj-domE*	SOJ2070
S3A	*Pom1-GFP::ura4+::kan^R ura4sj-D3 ade6sj-domE h+*	SOJ1339

3A	*pom1Δ::ura4+ ura4sj-D3 ade6sj-domE h+*	SOJ1031
3B, 4A	*Mid1-GFP::ura4+::kan*^{R} *Nhp6-mCherry::ura4+ ura4sj-D3 ade6sj-domE?*	SOJ1362
3B, 4B	*pom1Δ::ura4+ Mid1-GFP::ura4+::kan*^{R} *Nhp6-mCherry::ura4+ ura4sj-D3 ade6sj-domE?*	SOJ1400
3B, 3E	*Cdc15-GFP::ura4+::kan*^{R} *Pcp1-mCherry::ura4+::kan*^{R} *ura4sj-D3 ade6sj-domE*	SOJ2067
3B	*pom1Δ::ura4+ Rlc1-GFP::kan*^{R} *pAct1-Lifeact-mCherry::ura4+ ura4sj-D3 ade6sj-domE?*	SOJ1102
	pom1Δ::ura4+ Cdc15-GFP::ura4+::kan^R *Pcp1-mCherry::ura4+::kan*^{R} *ura4sj-D3 ade6sj-domE*	SOJ2161
3C, S3E	*pom1Δ::ura4+ Rlc1-GFP::kan*^{R} *Nhp6-mCherry::ura4+ ura4sj-D3 ade6sj-domE?*	SOJ1103
3D, S3F	*pom1Δ::ura4+ mid1Δ::ura4+ Rlc1-GFP::kan*^{R} *Nhp6-mCherry::ura4+ ura4sj-D3 ade6sj-domE?*	SOJ1586
3E	*pom1Δ::ura4+ mid1Δ::ura4+ Cdc15-GFP::ura4+::kan*^{R} *Pcp1-mCherry::ura4+::kan*^{R} *ura4sj-D3 ade6sj-domE*	SOJ2147
S3C	*mCherry-Rng2::ura4+ ura4sj-D3 ade6sj-domE h+*	SOJ1624
	pom1Δ::ura4+ mCherry-Rng2::ura4+ ura4sj-D3 ade6sj-domE	SOJ1658
	Cdr2-GFP::ura4+ ura4sj-D3 ade6sj-domE h+	SOJ974
	pom1Δ::ura4+ Cdr2-GFP::ura4+ ura4sj-D3 ade6sj-domE	SOJ1088
S3G	*pom1Δ::ura4+ Cdc15-GFP::ura4+::kan*^{R} *pAct1-Lifeact-mCherry::ura4+ Pcp1-mCherry::ura4+::kan*^{R} *ura4sj-D3 ade6sj-domE*	SOJ2162
4A	*GFP-Mid1*$^{S.p.}$*::ura4+::kan*^{R} *Nhp6-mCherry::ura4+ ura4sj-D3 ade6sj-domE?*	SOJ1449
4B	*pom1Δ::ura4+ GFP-Mid1*$^{S.p.}$*::ura4+::kan*^{R} *Nhp6-mCherry::ura4+ ura4sj-D3 ade6sj-domE?*	SOJ1447
4C, 4D, S4F	*pom1Δ::ura4+ GFP-Mid1*$^{S.p.}$*::ura4+::kan*^{R} *Rlc1-mCherry::ura4+::kan*^{R} *Pcp1-mCherry::ura4+::kan*^{R} *ura4sj-D3 ade6sj-domE*	SOJ1959
S4A	*GFP-Mid1*$^{S.p.}$*::ura4+::kan*^{R} *pAct1-Lifeact-mCherry::ura4+ ura4sj-D3 ade6sj-domE*	SOJ1451
S4B, S4C	*GFP-Mid1*$^{S.p.}$*::ura4+::kan*^{R} *Rlc1-mCherry::ura4+::kan*^{R} *Pcp1-mCherry::ura4+::kan*^{R} *ura4sj-D3 ade6sj-domE?*	SOJ1726
Schizosaccharomyces pombe		
**Figure**	**Genotype**	**Collection No.**
1A, S1A	*Rlc1-GFP::ura4+ Pcp1-mCherry::ura4+*	From M. Balasubramanian
S1B	*Rlc1-3GFP::kan*^{R} *pAct1-LifeAct-mCherry::leu1+*	From M. Balasubramanian
S1C	*pAct1-LifeAct-GFP::leu1+ Pcp1-mCherry::ura4+ leu1-32*	From M. Balasubramanian
1E	*Mid1-GFP::ura4+ Pcp1-mCherry::ura4+ ura4-D18 leu1-32 ade6*	SO3573
S3A	*Pom1-GFP::kan*^{R}	SO4594
S3D	*Cdc15-GFP::kan*^{R} *pAct1-LifeAct-mCherry::leu1+*	SO7955
	pom1Δ::ura4+ Cdc15-GFP::kan^R *pAct1-LifeAct-mCherry::leu1+*	SO7952
S4E	*ade6-M210 leu1-32 ura4-D18 h-*	SO1082
	mid1 Δ ::ura4+ leu1-32 ade6-M2x ura4-D18 h-	SO213
	GFP-Mid1$^{S.j.}$*::his5+::ura4+::mid1 leu1-32 ade5D ade7::Ade5 ura4-D18 his5D*	SO7810

Supplemental Experimental Procedures

Sequences and construction of strains

Fission yeast protein sequences were retrieved from the *Schizosaccharomyces* group database at the Broad Institute, USA (http://www.broadinstitute.org/annotation/genome/schizosaccharomyces_group/MultiHome.html) [S1]. Sequence alignment was performed using ClustalW2 [S2]. Phylogenetic trees were generated using Phylogeny (http://www.phylogeny.fr/) [S3]. To construct the LifeAct-GFP and LifeAct-mCherry *S. japonicus* strains, 1-kb PCR fragment of the *act1* promoter from *S. japonicus* was digested with ApaI and XhoI, and ligated into a pJK210-based pSO550 plasmid carrying the *S. japonicus ura4* gene as the selection marker. Nucleotide sequence encoding the LifeAct peptide was PCR amplified from the pJK148-Pact1-LAGFP plasmid, described in [S4]. LifeAct PCR fragment was joined to GFP or mCherry by fusion PCR. The LifeAct-GFP or LifeAct-mCherry fragment was double digested by XhoI and NotI and ligated into the pSO550 plasmid carrying the *act1* promoter. The final constructs pSO550-pAct1$^{S.japonicus}$-LifeAct-GFP/ mCherry were linearized with AfeI and transformed into yeast.

To generate gene knock-out strains in *S. japonicus*, we used either PCR-based homologous recombination strategy or the plasmid-based methodology. Briefly, primers harboring 80-bp flanking sequence of the target gene were used to amplify the KanMX6 cassette as the selection marker. The purified PCR product was directly used for yeast transformation. Alternatively, a pSO550 plasmid carrying the 5'UTR and 3'UTR fragments of the target gene, each approximately 500-bp in length, was linearized by appropriate enzymes and used for yeast transformation.

To generate the C-terminally tagged Rlc1-GFP strain, we used the PCR-based homologous recombination strategy as described in [S5]. Pcp1-mCherry, Mid1-GFP, Pom1-GFP, Cdc15-GFP, Cdc15-mCherry and Rlc1-mCherry were constructed according to the strategy described in [S6] with some modifications. Briefly, BglII- and NheI-digested PCR fragment of the KanMX6 cassette was used to replace the *S. japonicus ura4* cassette with the same flanking restriction enzyme sites as in the pSO550 plasmid. This construct was digested by BamHI and XbaI and ligated with the *S. japonicus ura4* cassette harboring the first 687 bp of *S. japonicus ura4* ORF. The resulting plasmid pKanMX6-Ura4ΔC$^{S. japonicus}$ was used to integrate the sequence encoding a C-terminally deleted *S. japonicus* Ura4 protein at the 3'UTR region of any target gene (creating an acceptor strain). A second pBluescriptKS-based plasmid was constructed to place the DNA sequence encoding a missing C-terminal fragment of S. japonicus Ura4 between BamHI and XbaI, to make the pKS-Ura4C$^{S. japonicus}$. DNA sequences encoding the C-terminus of any target protein fused to GFP or mCherry were cloned into pKS-Ura4C$^{S. japonicus}$ using ApaI and ClaI. The protein coding sequences were followed by ~200-bp 3'UTR of the target genes inserted between SmaI and BamHI sites. The resulting plasmid was used to PCR-amplify the region encoding the C-terminal target gene sequence followed by the fluorophore, the 3'UTR of the target gene and the Ura4 C-terminal fragment. This PCR product was used to transform the acceptor strain. Transformants were selected on the minimal medium plates without uracil. The GFP-Mid$^{S.pombe}$ *S.japonicus* strain was constructed similarly. As a result, the *S. japonicus mid1* promoter drove the expression of the N-terminally GFP-tagged *S. pombe* Mid1 that replaced the *S. japonicus mid1* ORF at its native locus.

To construct the N-terminally GFP- or mCherry-tagged Rng2 and Myo2 strains, the corresponding promoter sequences of *rng2* or *myo2* genes flanked by KpnI and ApaI sites were ligated into pSO550 plasmid carrying the *S. japonicus ura4* cassette. The obtained plasmids were then ligated to GFP- or mCherry-encoding sequences flanked by ApaI and XhoI, followed by the N-terminal ORF of Rng2 or Myo2 using XhoI and SmaI, and the adjacent genomic DNA fragments upstream to the promoter regions flanked by SmaI and BamHI. The obtained N-tagging plasmids were linearized by SmaI and transformed into yeast.

To create temperature sensitive mutants of *cdc25-D9* and *cdc15-A,* a PCR-based random mutagenesis strategy was adapted from [S6] with some modifications mentioned above. Acceptor strains carrying the C-terminally truncated *ura4* ORF cassette at the 3'UTR region of either *cdc25* or *cdc15* were generated using the plasmid pKanMX6-Ura4ΔC$^{S. japonicus}$. Error-prone PCR products were amplified using the template plasmid pKS-Ura4C$^{S. japonicus}$ harboring a sequence of interest and used to transform the acceptor strain to reconstitute the *ura4+* cassette as the selective marker for successful integration. *cdc25-D9* carries four mutations in the ORF that change amino acid residues at positions 30 (Ile to Thr), 446 (Ile to Val), 494 (Thr to Ala) and 572 (Thr to Pro). *cdc15-A* allele carries two mutations in the encoded protein at positions 264 (Leu to Pro) and 442 (Pro to Thr).

To generate Mid1-NLS^{SV40}-GFP, a nucleotide sequence encoding the NLS (PKKKRKV) from the SV40 large T-antigen was inserted between codons corresponding to the amino acid position 725 and 726 of *S. japonicus mid1* ORF by fusion PCR. To construct Mid1-NLS$^{S.p.}$ –GFP, nucleotide sequence of *S. japonicus mid1* encoding amino acid residues from 650 to 760 was replaced with a coding region of *S. pombe mid1* spanning amino acid residues 639 to 730 by fusion PCR. To obtain Mid1-Δ$^{711\text{-}723a.a.}$-GFP, a nucleotide sequence encoding the amino acid residues 711 to 723 of *S. japonicus mid1* ORF was deleted via fusion PCR.

mid1/mid2 transcription during cell cycle

Cell cycle synchronization of *S. japonicus* was performed using *cdc25-D9* conditional mutant. Briefly, *cdc25-D9* cells were grown in YES medium at 24°C to OD_{595} 0.2 and shifted to 36°C for 3 hours 30 minutes. Cells were collected and snap frozen at G2/M transition block point and at 20-minute intervals after lowering the temperature to 24°C. Total RNA was extracted using TRIzol® Reagent (Life Technologies). Transcriptor First Strand cDNA Synthesis Kit (Roche diagnostics) was used to synthesize cDNAs with anchored-oligo(dT)18 primer. PCR primers for real-time quantitative PCR (*mid1* 5'-TCCTGCGACAACTTCACCTC-3', 5'-GGCTTCCACCTGCTTCTCAT-3'; *mid2* 5'- CCATTCGCTCTTTCGGCATG-3', 5'- AGGTGTGTTGGCATCGTAGG-3'; *hcs1* 5'-TTTTCGAGCCTGGTATGCGT-3', 5'-CAGTCAAGGTTTGCTCACGC-3') were designed using Primer3 (Whitehead Institute, United States). Real-time quantitative PCR was performed with FastStart Essential DNA Green Master (Roche diagnostics). Data was collected and analyzed with LightCycler® 96 System (Roche diagnostics). *mid1/mid2* transcript levels were shown as relative linear ratio after normalization to *hcs1.*

Microscope image acquisition and data analyses

For time-lapse movies, mid-log phase cells were concentrated by centrifugation at 3,000 rpm for 30 sec and imaged on 2% agarose pads. Most imaging experiments were performed at 24°C unless indicated otherwise. Epi-fluorescence images in Fig. 3A, S1D and S4E were captured using a Zeiss AxioVert200M microscope (Carl

Zeiss; Plan Apochromat 100×/1.45 NA oil objective lens) equipped with the CCD camera (CoolSNAP HQ; Photometrics) and driven by MetaMorph-v6.2r6 software package (Molecular Devices). Zeiss Axio Observer Z1 fluorescence microscope fitted with α Plan-FLUAR 100x/1.45 NA oil objective lens (Carl Zeiss) and the Orca-Flash4.0 C11440 camera (Hamamatsu) was used to acquire images shown in Fig. 2B, S3D, G and S4C. Scanning confocal images in Fig. 1C and S1B were acquired using a Nikon A1R Si Confocal system with Eclipse Ti-E Inverted microscope, Nikon Plan Apo VC 100x/NA1.4 oil objective, 457nm/488nm/514nm Argon, 561nm diode lasers, GaAsP Detectors, driven by NIS Elements C Scanning confocal images in Fig. 1E, 3C, S1(H, I), S2(B, C) and S3A were acquired using LSM510 microscope equipped with a Plan Apochromat 100X, 1.4 N.A. objective lens, a 488-nm argon laser and a 543-nm HeNe laser. Spinning-disk confocal images in Fig. 1(A, B and D), 3(C and D), 4(A-D), S1(A, C), S3(E, F). S4(A, B, F) were captured by the microLAMBDA system built on Eclipse Ti Nikon inverted microscope (Plan Apochromat VC 100×/1.40 NA oil objective lens) and equipped with CSUX1FW spinning disk head (Yokogawa Corporation of America), CCD camera (CoolSNAP HQ2), under the control of MetaMorph-v7.7.7.0 software. A 491-nm diode-pumped solid-state (DPSS) laser (Calypso) and 561-nm DPSS laser (Jive) were used for excitation. Spinning-disk confocal images in Fig. 2A, Fig. 3E and S2D were captured by Yokogawa CSU-X1 Spinning Disk Confocal with Eclipse Ti-E Inverted microscope with Nikon Plan Apo VC 100x/NA1.4 oil objective, 488nm diode, 561nm diode lasers and Andor Ixon3 EM-CCD camera controlled by NIS Elements AR. 0.5-μm step size images were typically collected.

When required, images were analyzed using ImageJ (National Institutes of Health). Ring assembly time was measured by time points spent between SPBs separation labeled by Pcp1-mCherry and fully formation of the actomyosin ring marked by Rlc1-GFP. Ring constriction time was shown as time points after the appearance of a fully formed ring till it completes constriction. Average ring assembly or constriction time was shown as mean±standard deviation. Kymographs were made with ImageJ plug-in KymoResliceWide developed by Eugene Katrukha.

Measurements of fluorescence intensity

Spinning-disk confocal images of Z-stacks with 0.5μm interval were processed with ImageJ using the sum projection method. For fluorescence intensity measurement of Rlc1-GFP, the mean intensity of an ROI created within any cellular boundary of the projected image was obtained and the background was background-subtracted. Fluorescence intensity was shown as mean±standard deviation.

Myosin nodes tracking

Time-lapse single plane images (10 sec/frame, 730 frames in total) of interphase wt and *pom1Δ* cells, carrying the myosin II light chain Rlc1-GFP and the SPB marker Pcp1-mCherry acquired with Zeiss observer epifluorescence microscope, were processed with TrackMate v2.3.0 (Fiji). Briefly, images were background-subtracted and then filtered using LoG detector, followed by LAP tracking with gap-closing, track splitting and merging functions. Tracking profile was further analyzed with Icy v1.5.2.0 (Quantitative Image Analysis Unit, Institut Pasteur) to obtain measurements shown in Fig. S3B. Only nodes that can be tracked progressively more than three consecutive frames were included.

LatrunculinA treatment

S. japonicus cells were treated with 10 μM LatrunculinA (Biomol International LP) for 15 minutes before imaging.

Cell centrifugation

All centrifugation experiments were done with a microcentrifuge (Heraeus Pico 17, Thermo Scientific) for 2 min at 13,300 rpm before imaging.

Supplemental References

S1. Rhind, N., Chen, Z., Yassour, M., Thompson, D.A., Haas, B.J., Habib, N., Wapinski, I., Roy, S., Lin, M.F., Heiman, D.I., et al. (2011). Comparative functional genomics of the fission yeasts. Science *332*, 930-936.

S2. Larkin, M.A., Blackshields, G., Brown, N.P., Chenna, R., McGettigan, P.A., McWilliam, H., Valentin, F., Wallace, I.M., Wilm, A., Lopez, R., et al. (2007). Clustal W and Clustal X version 2.0. Bioinformatics *23*, 2947-2948.

S3. Dereeper, A., Guignon, V., Blanc, G., Audic, S., Buffet, S., Chevenet, F., Dufayard, J.F., Guindon, S., Lefort, V., Lescot, M., et al. (2008). Phylogeny.fr: robust phylogenetic analysis for the non-specialist. Nucleic Acids Res *36*, W465-469.

S4. Huang, J., Huang, Y., Yu, H., Subramanian, D., Padmanabhan, A., Thadani, R., Tao, Y., Tang, X., Wedlich-Soldner, R., and Balasubramanian, M.K. (2012). Nonmedially assembled F-actin cables incorporate into the actomyosin ring in fission yeast. J Cell Biol *199*, 831-847.

S5. Bahler, J., Wu, J.Q., Longtine, M.S., Shah, N.G., McKenzie, A., 3rd, Steever, A.B., Wach, A., Philippsen, P., and Pringle, J.R. (1998). Heterologous modules for efficient and versatile PCR-based gene targeting in Schizosaccharomyces pombe. Yeast *14*, 943-951.

S6. Tang, X., Huang, J., Padmanabhan, A., Bakka, K., Bao, Y., Tan, B.Y., Cande, W.Z., and Balasubramanian, M.K. (2011). Marker reconstitution mutagenesis: a simple and efficient reverse genetic approach. Yeast *28*, 205-212.
